# Light at night exposure and risk of dementia conversion from mild cognitive impairment in a Northern Italy population

**DOI:** 10.1186/s12942-024-00384-5

**Published:** 2024-11-23

**Authors:** Tommaso Filippini, Sofia Costanzini, Annalisa Chiari, Teresa Urbano, Francesca Despini, Manuela Tondelli, Roberta Bedin, Giovanna Zamboni, Sergio Teggi, Marco Vinceti

**Affiliations:** 1 Environmental, Genetic and Nutritional Epidemiology Research Center (CREAGEN), Department of Biomedical, Metabolic and Neural Sciences, Section of Public Health, University of Modena and Reggio Emilia, 287 Via Campi, Modena, 41125 Italy; 2 School of Public Health, University of California Berkeley, Berkeley, CA USA; 3 DIEF - Department of Engineering ‘Enzo Ferrari’, University of Modena and Reggio Emilia, Modena, Italy; 4 Neurology Unit, University Hospital of Modena, Modena, Italy; 5 Department of Biomedical, Metabolic, and Neural Sciences, University of Modena and Reggio Emilia, Modena, Italy; 6 Department of Epidemiology, Boston University School of Public Health, Boston, MA USA

**Keywords:** Alzheimer’s dementia, Dementia, Environmental factors, Light at night, Mild cognitive impairment, Risk

## Abstract

**Background:**

A few studies have suggested that light at night (LAN) exposure, i.e. lighting during night hours, may increase dementia risk. We evaluated such association in a cohort of subjects diagnosed with mild cognitive impairment (MCI).

**Methods:**

We recruited study participants between 2008 and 2014 at the Cognitive Neurology Clinic of Modena Hospital, Northern Italy and followed them for conversion to dementia up to 2021. We collected their residential history and we assessed outdoor artificial LAN exposure at subjects’ residences using satellite imagery data available from the Visible Infrared Imaging Radiometer Suite (VIIRS) for the period 2014–2022. We assessed the relation between LAN exposure and cerebrospinal fluid biomarkers. We used a Cox-proportional hazards model to compute the hazard ratio (HR) of dementia with 95% confidence interval (CI) according to increasing LAN exposure through linear, categorical, and non-linear restricted-cubic spline models, adjusting by relevant confounders.

**Results:**

Out of 53 recruited subjects, 34 converted to dementia of any type and 26 converted to Alzheimer’s dementia. Higher levels of LAN were positively associated with biomarkers of tau pathology, as well as with lower concentrations of amyloid β_1−42_ assessed at baseline. LAN exposure was positively associated with dementia conversion using linear regression model (HR 1.04, 95% CI 1.01–1.07 for 1-unit increase). Using as reference the lowest tertile, subjects at both intermediate and highest tertiles of LAN exposure showed increased risk of dementia conversion (HRs 2.53, 95% CI 0.99–6.50, and 3.61, 95% CI 1.34–9.74). In spline regression analysis, the risk linearly increased for conversion to both any dementia and Alzheimer’s dementia above 30 nW/cm^2^/sr of LAN exposure. Adding potential confounders including traffic-related particulate matter, smoking status, chronic diseases, and apolipoprotein E status to the multivariable model, or removing cases with dementia onset within the first year of follow-up did not substantially alter the results.

**Conclusion:**

Our findings suggest that outdoor artificial LAN may increase dementia conversion, especially above 30 nW/cm^2^/sr, although the limited sample size suggests caution in the interpretation of the results, to be confirmed in larger investigations.

**Supplementary Information:**

The online version contains supplementary material available at 10.1186/s12942-024-00384-5.

## Background

Dementia is a progressive cognitive syndrome with increasing incidence in the decades. It is expected that subjects with dementia could nearly triple from 57 million in 2019 up to over 150 million in 2050 [1]. Some non-modifiable risk factors including aging, female sex, and genetic susceptibility are known to increase disease risk [2–5]. However, a pivotal role of environmental and lifestyle factors has been suggested for dementia onset and progression [5–7]. These include low education and socio-economic status, dietary habits, traumatic brain injury, and cardiovascular diseases independently from stroke [8–15].

Environmental factors linked to urbanization and climate change have been recently outlined [16, 17], especially outdoor air pollution [18–21], noise pollution [22, 23], greenness [24–27]. In addition, outdoor artificial lighting during night hours, generally referred as “light at night” (LAN), has been pointed out as possible relevant linkage within the relation between urban environment and human health [28–31].

LAN exposure already demonstrated detrimental effects in humans due to different mechanisms, mainly disruption of circadian rhythm, sleep disturbances, and altered secretion of melatonin and other hormones [32–34]. Among the investigated health outcomes, LAN exposure has been reported to increase risk of some types of cancer [35, 36], metabolic diseases [37–39], and mental disorders [40–42].

Findings from few experimental animal and laboratory studies indicates that light exposure during night cycle and disruption of circadian rhythm may affect neuron vitality and activity through several mechanisms including tau protein deposition, altered neuronal architecture and increased oxidative stress [43–50]. In addition, two recent epidemiologic studies reported a positive relation between LAN and risk of dementia [51, 52]. However, evidence is still scarce.

For these reasons, we aimed to evaluate the association between LAN exposure and dementia risk of conversion to dementia in an Italian population of subjects diagnosed with mild cognitive impairment (MCI).

## Methods

### Study population

We recruited subjects who had been diagnosed with mild cognitive impairment (MCI) during 2008–2014 period at the Cognitive Neurology Clinic of Modena University-Hospital in the province of Modena, Northern Italy [53]. The study was carried out following the principles of the Helsinki declaration and received approval by the Modena Ethics Committee (no. 84/2015). MCI diagnosis was given according to Peterson’s criteria and included amnestic (single or multiple domain) or non-amnestic forms of MCI [54, 55]. In addition to a diagnosis of MCI, inclusion criteria also comprised availability of cerebrospinal fluid (CSF) sample for analysis, and residence in the Modena province. Study flow-chart for subject identification and selection is reported in Supplementary Figure S1.

During the diagnostic assessment, all subjects had undergone clinical neurological evaluation, including brain imaging examinations and neuropsychological assessment. We performed lumbar puncture to collect a CSF sample for biomarker assessment [56, 57]. We collected demographic information and other socio-demographic characteristics, including sex, date and place of birth, education, residential history, smoking habits and comorbidities. We also assessed apolipoprotein E ε4 (APOE4) genotype using real-time PCR [58].

We followed-up cohort participants with six-monthly assessments until August 2021 [59]. During follow-up visits, subjects were classified as non-converters or as converters to any form of dementia, including subdivision in main categories: Alzheimer’s dementia (AD), frontotemporal dementia (FTD), Lewy-body dementia (LBD), and vascular dementia. Diagnoses were all revised *a posteriori* by expert neurologists (AC, MT, GZ) in order to harmonize the classification according to the most recent diagnostic criteria [60–63].

### CSF biomarker assessment

We collected and processed CSF samples according to standard procedures and quantified amyloid β 1–42 (amyloid Aβ_1−42_), total (t-tau), and phosphorylated tau (p-tau181) proteins (INNOTEST, Innogenetics, Belgium) as previously reported in details [64]. In summary, we performed sample collection via lumbar puncture in fasting subjects using the Standard International Procedures for CSF Biobanking [65]. We transferred and processed CSF samples at the Modena Neuroimmunology Laboratory, within 30 min from collection. Samples were anonymized with an alphanumeric code and laboratory personnel was blinded to subjects’ identity and clinical data. Each sample was centrifuged at controlled room temperature for 10 min at 2500 *g* for 10 min. If analytical determination could not be immediately performed after centrifugation, samples were aliquoted into polypropylene sterile tubes and stored at -80 °C until testing.

### Exposure assessment to environmental factors

We collected residential history through access of personal data and interview performed by one neurologist of the team. We assessed exposure to outdoor artificial LAN through geocoding addresses of residence at the year of recruitment and we checked that it did not change in the former 5 years. We geocoded residential data using Google Earth Pro software and OpenStreetMap website considering the coordinates of the centroid of the house of residence of the subjects. We used satellite imagery data available from the Visible Infrared Imaging Radiometer Suite (VIIRS) provided by the National Aeronautics and Space Administration (NASA) 2011 mission through the Suomi National Polar-Orbiting Partnership (Suomi NPP) spacecraft [66].

We used the Global Nighttime light maps elaborated by the Earth Observation Group (EOG) of the Colorado School of Mine’s Payne Institute. Specifically, we used the VIIRS Stray Light Corrected Nighttime Day/Night Band Composites Version 1 (imagery of the Google Earth Engine Data Catalog). This dataset consists in composite radiance images of nighttime data from the VIIRS Day/Night Band (DNB) and we calculated monthly average. This product excludes data affected by cloud cover and uses a procedure to correct imagery for stray light. Products are available for the period 2012–2022 (spatial resolution of 15 arc second - RS WGS 84 latitude/longitude). To provide an indicator of the exposure to artificial lights for the investigated subjects, expressed in radiance (nW/cm^2^/sr), we implemented a Google Earth Engine procedure considering imagery from 2014 to 2022, centered in the follow-up period of the patients. Starting from monthly imagery, the procedure calculated annual average and then extracted for each subject the corresponding annual radiance value.

In order to evaluate and select individual LAN exposure data, we calculated the 9-year average for the entire follow-up-period 2014–2022 and run matrix analysis to assess the correlation across different yearly VIIRS data and overall period. As individual LAN exposure values, we considered the 9-year average VIIRS data for the 2014–2022 period in the main analysis, and the mean annual VIIRS data for 2014 in the sensitivity analysis. We considered that specific 2014 year because it is the first available and closest to recruitment period. Finally, we assessed subjects’ exposure to traffic-related concentrations of particulate matter with diameter < 10 μm (PM_10_) using a validated air dispersion model (CALINE4) as previously described [67–69]. For the purpose of this analysis, we used mean annual PM_10_ concentrations.

### Data analysis

We assessed annual median values (along with interquartile range-IQR) of outdoor artificial LAN exposure for overall subjects and their characteristics, namely sex, age (divided into < 65 and ≥ 65 years at recruitment), education (< 8 years, 8–12 years, and ≥ 13 years), and smoking habits. We implemented a restricted cubic spline regression model with three knots at fixed (10, 50, and 90) percentiles to evaluate the relation between LAN exposure and CSF baseline levels of the biomarkers of amyloidosis (amyloid Aβ_1−42_), tau pathology (p-tau181) and neurodegeneration (t-tau) [70]. In the main analysis, we used an unadjusted model, while in sensitivity analysis we further adjusted by traffic-related PM_10_ mean concentrations due to possible confounding based on our previous study [71] and similar recent evidence [72, 73].

We then assessed risk of dementia conversion by computing hazard ratio (HRs) with 95% confidence intervals (CIs) through a Cox proportional hazards model. We calculated person-time at risk as the time ranging from date of MCI diagnosis and dementia diagnosis, or end of follow-up (August 31, 2021), whichever occurred first. We considered as possible outcome of interest incidence of any type of dementia as well as of AD only, this latter after exclusion of subjects with subsequent diagnosis of other dementia types from the entire analysis. Such analysis could not be implemented for the other dementia forms due to too low number of subjects with other diagnosis.

We run the model using LAN exposure as both continuous (1-unit and 10-unit increase), and categorical variable with three different methods: (i) < median and ≥ median value; (ii) by tertiles; (iii), by fixed cut-offs at values < 15 nW/cm^2^/sr, ≥ 15 but < 30 nW/cm^2^/sr, and ≥ 30 nW/cm^2^/sr. Finally, we assessed possible non-linear relation using restricted cubic spline within the Cox regression model at fixed percentiles (10th, 50th and 90th).

We checked proportionality assumption for the investigated independent variables. Since it was violated for sex, the main adjusted model was stratified by sex (males/females) and adjusted by age at entry (continuous values), and education (three categories: <8, 8–12, and ≥ 13 years). We also run sensitivity analysis further adjusting alternatively for smoking habits, mean traffic-related PM_10_ concentrations, chronic obstructive pulmonary disease (COPD), diabetes, and apolipoprotein E status, although this latter was missing for 15 participants. Finally, we performed analysis excluding smokers, and also individuals converting to dementia within one year from the date of recruitment. We used Stata v.18 (StataCorp., College Station, TX, 2023) for all data analysis using ‘mkspline’, ‘stset’, and ‘stcox’ routines.

## Results

Study population characteristics at baseline are reported in Table 1. Overall, we recruited 53 (males/females: 28/25) subjects with MCI with available CSF samples and with available environmental data. Median age at recruitment was 66.3 years (range 42.6–81.6 years), slightly higher in males (67.1 years) than females (64.7 years).

**Table 1:** Baseline characteristics of study participants with mild cognitive impairment (MCI) divided according to diagnosis at the end of follow-up: MCI not converted, any dementia, or Alzheimer’s dementia (AD). Number of overall subjects in each category reported in parenthesis. Median and interquartile range (IQR) levels visible infrared imaging Radiometer Suite (VIIRS) data for 9-year average 2014–2022 period of outdoor artificial light at night (LAN) exposure in nW/cm^2^/sr for the overall population are reported. Number of overall subjects in each category reported in parenthesis

	MCI not converted		Any dementia		AD		LAN exposure 2014–2022
	*N*	**%**	*N*	**%**	*N*	**%**	**Median (IQR)**
All subjects (*n* = 53)	19	100	34	100	26	100	26.2 (14.9–33.8)
Sex							
Males (*n* = 28)	11	57.9	17	50.0	12	46.1	26.2 (14.1–34.1)
Females (*n* = 25)	8	42.1	17	50.0	14	53.9	26.4 (16.9–32.8)
Age at first diagnosis							
< 65 years (*n* = 22)	10	52.6	12	35.3	8	30.8	25.6 (14.5–32.8)
≥ 65 years (*n* = 31)	9	47.4	22	64.7	18	69.2	26.5 (15.7–34.3)
Educational attainment							
< 8 years (*n* = 18)	9	47.4	9	26.5	7	26.9	31.4 (23.1–36.0)
8–12 years (*n* = 15)	3	15.8	12	35.3	10	38.5	26.1 (14.4–33.0)
≥ 12 years (*n* = 20)	7	36.8	13	38.2	9	34.6	24.7 (11.2–31.7)
Smoking habits							
Non-smokers (*n* = 46)	18	94.7	28	82.4	21	80.8	26.2 (13.6–33.8)
Smokers (*n* = 7)	1	5.3	6	17.6	5	19.2	26.2 (25.4–34.4)
COPD							
No (*n* = 51)	19	100	32	94.1	25	96.2	26.4 (14.5–34.3)
Yes (*n* = 2)	0	0.0	2	5.9	1	3.8	25.8 (25.4–26.1)
Diabetes							
No (*n* = 51)	18	94.7	33	97.1	25	96.2	26.2 (14.9–34.3)
Yes (*n* = 2)	1	5.3	1	2.9	1	3.8	23.6 (14.5–32.8)
APOE4 genotype							
Negative (*n* = 21)	10	52.6	11	32.3	6	23.1	25.5 (13.6–34.4)
Positive (*n* = 17)	3	15.8	14	41.2	12	46.1	31.1 (16.9–33.0)
Missing (*n* = 15)	6	31.6	9	26.5	8	30.8	25.7 (23.1–32.8)
Follow-up (months)^a^	93	58–110	47	36–59	47	36–65	-

^a^Values are median and interquartile range. Abbreviations: AD, Alzheimer’s dementia; APOE4, apolipoprotein E ε4 genotype; COPD, chronic obstructive pulmonary disease; IQR: interquartile range; LAN: light at night; MCI: mild cognitive impairment, VIIRS: Visible Infrared Imaging Radiometer Suite

During an overall median follow-up of 54 months (IQR: 38–89 months), 19 subjects did not convert and MCI diagnosis was confirmed, while 34 subjects converted to any type of dementia, 26 of whom converted to AD. Among the non-converters, there was a higher number of subjects who were younger than 65 at recruitment, had lower education, and were non-smokers (Table 1). Two subjects were affected by COPD, both in those who converted. Similarly, two subjects were diagnosed with diabetes, one each in those converted and not-converted. Assessment of APOE4 genotype was available for 38 subjects, showing a higher percentage in subjects who converted to any dementia or AD. Details on other types of dementia are reported in Supplementary Table S1.

**Fig. 1:**
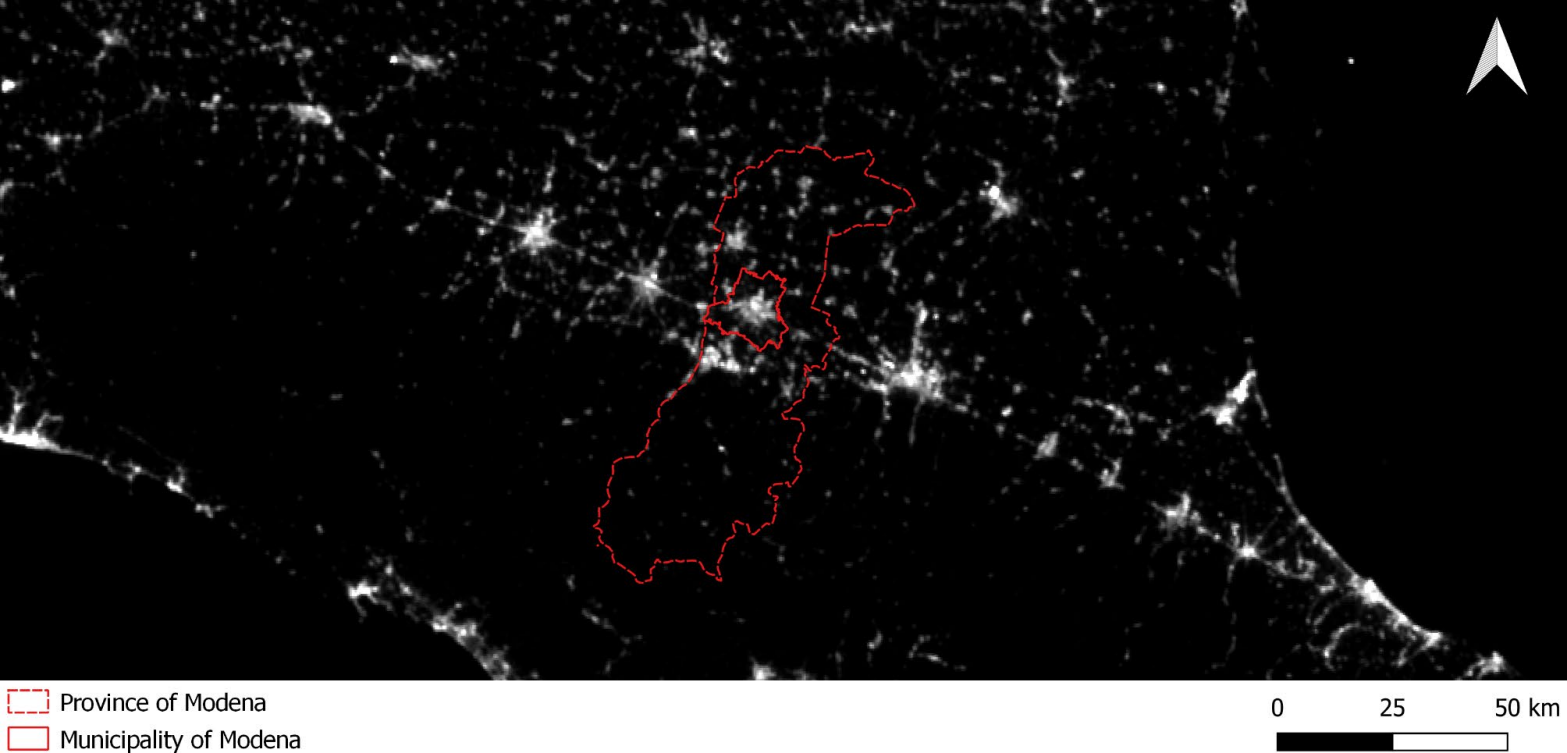
Map of light at night in the Emilia-Romagna region (Northern Italy) with indication of the study area of Modena province (dash red line) and municipality (solid red line) obtained using 9-year average 2014–2022 period of Visible Infrared Imaging Radiometer Suite (VIIRS) data

The 9-year 2014–2022 average annual VIIRS data over the study area is shown in Fig. 1. Median (IQR) level of annual LAN exposure of the 2014–2022 period is 26.4 nW/cm^2^/sr (IQR: 14.9–33.5) (Table 1). Levels are almost similar in males and females and with or without COPD, but slightly higher in subjects aged ≥ 65 years at recruitment, smokers, subjects with lower education (< 8 years), those without diabetes and those positive for APO4E genotype. Data are substantially comparable when considering yearly VIIRS data (Supplementary Table S2 and Figure S2). Correlation analysis showed very comparable values of VIIRS data across the considered years and with VIIRS 2014–2022 average (always > 0.95) (Supplementary Table S3 and Figures S3).

**Fig. 2:**
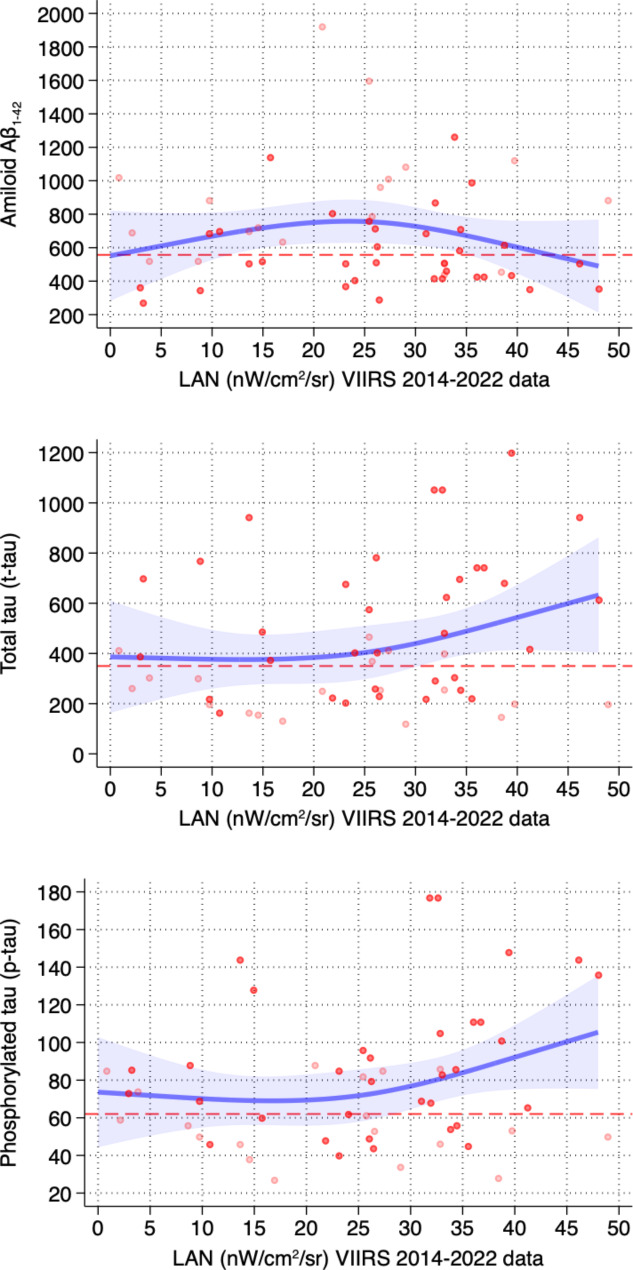
Spline correlation analysis between outdoor artificial light at night (LAN) exposure and levels of cerebrospinal biomarkers, i.e. beta-amyloid, total tau protein, phosphorylated tau-protein. Light and dark red dots indicate subjects who remained MCI and converted to dementia, respectively. The blue line represents spline regression analysis with 95% confidence interval (light blue area). The red continuous line represents the biomarker cut-off values used at the Modena Neuroimmunology Laboratory (amyloid Aβ_1−42_: 557 pg/mL; t-tau: 350 pg/mL; p-tau181: 62 pg/mL)

In the restricted cubic splines, there is an almost null association between LAN exposure till 30 nW/cm^2^/sr and baseline concentrations of biomarkers (Fig. 2). Above this level, there is a strong positive association with p-tau181 and t-tau and a slightly inverse association with amyloid Aβ_1−42_. Further adjustment for mean PM_10_ concentrations marginally affected the relations (Supplementary Figure S4).

Distribution of subjects according to increasing outdoor artificial LAN exposure and risk of conversion to any type of dementia and AD are reported in Table 2. Linear increase of LAN exposure shows positive association with HR of 1.04 (95% CI 1.01–1.07). Using the lowest tertile as reference category, LAN exposure in the second and third tertiles shows dose-response increase with HRs of 2.53 (95% CI 0.99–6.50) and 3.61 (95% CI 1.34–9.74), respectively. Similar trends are depicted by division with fixed categories. Similar increase in risk can be noted in the analysis restricted to conversion to AD, although characterized by higher imprecision due to lower number of subjects. Further adjustments for relevant confounders confirm such increase in risk, with especially stronger association after controlling for traffic-related PM_10_ concentrations, smoking status, COPD and diabetes (Supplementary Tables S4-S7), and to a lesser extent also for APOE4 status (Supplementary Table S8). Analysis restricted to non-smokers shows similar results to the overall population (Supplementary Table S9), as it was the case after exclusion of subjects with diagnosis within 12 months (Supplementary Table S10). In the sensitivity analysis using 2014 VIIRS, we found almost identical results (Supplementary Tables S11-S18).

**Table 2:** Risk of dementia according to increasing exposure to outdoor artificial light at night (LAN) using 9-year average 2014–2022 period of VIIRS data, using both linear increase and categories based on median (50th), tertiles, and fixed cutoffs of nighttime luminance exposure (LAN) in nW/cm^2^/sr, in a multivariable analysis stratified by sex and adjusted by age, and educational attainment. Analysis considering as outcome any dementia, and Alzheimer’s dementia only (with exclusion of other dementia cases ab initio). HR: hazard ratio, CI: confidence interval

2014–2022				Any dementia					AD	
LAN (nW/cm^2^/sr)	50th	*N* C+/C-	OR	(95% CI)	*P* value	50th	*N* C+/C-	OR	(95% CI)	*P* value
Linear trend (1-unit increase)	-		1.04	(1.01–1.07)	0.021	-		1.01	(1.00-1.07)	0.052
Linear trend (10-unit increase)	-		1.44	(1.06–1.97)	0.021	-		1.40	(1.00-1.97)	0.052
**LAN-Median**										
Below the median	14.7	15/11	1.00	-		14.1	11/11	1.00	-	
Above or equal the median	33.8	19/8	1.83	(0.87–3.84)	0.109	33.8	15/8	1.90	(0.82–4.40)	0.137
**LAN-Tertiles**										
1st tertile	9.7	8/9	1.00	-		9.7	6/9	1.00	-	
2nd tertile	26.2	13/5	2.53	(0.99–6.50)	0.053	26.3	9/5	2.16	(0.72–6.50)	0.172
3rd tertile	36.4	13/5	3.61	(1.34–9.74)	0.011	36.4	11/5	3.62	(1.21–10.87)	0.022
**LAN - fixed cut-offs**										
< 15	9.2	7/7	1.00	-		8.8	6/7	1.00	-	
≥ 15; < 30	25.4	10/7	2.00	(0.72–5.60)	0.186	25.7	6/7	1.42	(0.44–4.56)	0.558
≥ 30	35.0	17/5	3.57	(1.32–9.70)	0.013	34.4	14/5	2.88	(1.02–8.15)	0.046

**Fig. 3:**
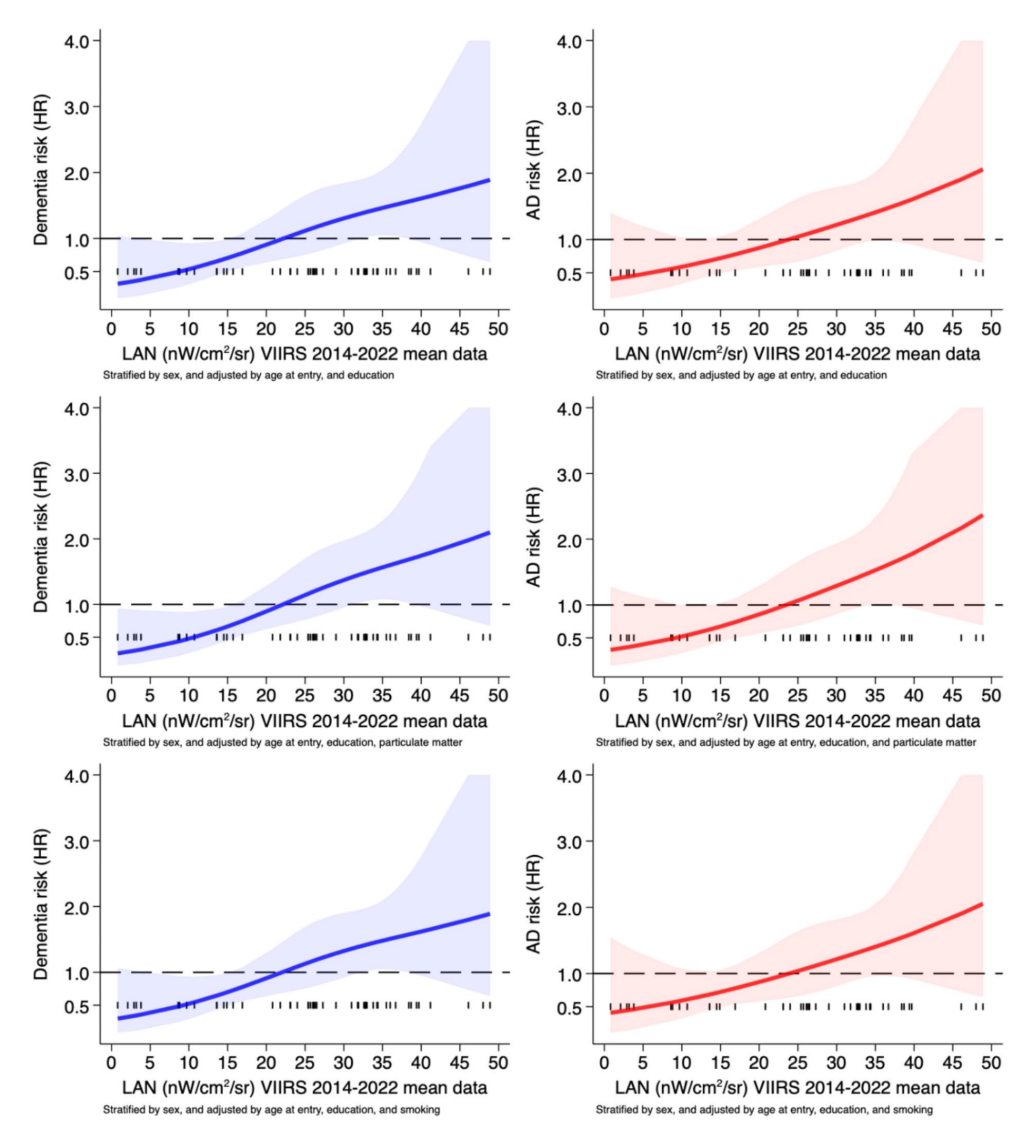
Spline regression analysis for the association between outdoor articificial light at night (LAN) using 9-year average 2014–2022 Visible Infrared Imaging Radiometer Suite (VIIRS) data and risk of developing any type of dementia. The solid line indicates hazard ratio (HR) and the shaded areas the 95% confidence intervals. Analysis considering as outcome any dementia (blue), and Alzheimer’s dementia only (red-with exclusion of other dementia cases ab initio). Analysis stratified by sex and adjusted by age at entry, and education, and further for particulate matter, or smoking status

Spline regression analysis assessing non-linear relation is reported in Fig. 3, showing substantial linear risk for both any type of dementia and AD above 30 nW/cm^2^/sr of LAN exposure. The relation does not change across adjusted models further accounting for PM_10_ concentrations and smoking status (Fig. 3). Alternative adjustment by COPD and diabetes shows similar results for any type of dementia and AD (Supplementary Figure S5). Adjustment by APO4E status yields similar results for any type of dementia, while almost null association can be noted for AD, with the risk starting to increase above 35–40 nW/cm^2^/sr only (Supplementary Figure S5). In the sensitivity analysis restricted to non-smokers (Supplementary Figure S6) and excluding subjects with diagnosis within 12 months (Supplementary Figures S7-S8), the relation is almost identical to the overall population, as it was the case when using 2014 VIIRS data instead of 2014–2022 average (Supplementary Figures S9-S13).

## Discussion

In our prospective study carried out in a Northern Italy population with MCI, we showed a positive association between outdoor artificial LAN exposure and increased risk of conversion to dementia. Such association appears to have a threshold effect above 30 nW/cm^2^/sr. It remained strong also after adjustment for relevant confounders including sex, age at entry, education, traffic-related air pollution and smoking history, and in sensitivity analyses performed after exclusion of subjects converted within 1 year and smokers. The association became almost linear when specifically focusing on conversion to AD.

Our findings are consistent with a previous study reporting a positive association between LAN exposure and MCI prevalence in Chinese veterans [51]. Conversely, in a study carried out in the same Italian area, we found contrasting results. Indeed, using a case-control design, LAN showed inconsistent association with early-onset dementia, while we found an almost linear positive association with late-onset dementia (LOD), although characterized by a high imprecision of the risk estimates [52].

Nonetheless, our results further support the hypothesis that either dementia onset or progression may be driven by non-genetic risk factors such as LAN, within the general landscape considering genetic and environmental risk factors and their complex interplay [7]. Interestingly, we reported consistent associations between LAN and dementia risk when assessing specific CSF biomarkers of neurodegeneration, specifically increased levels of t-tau and p-tau181 and decreased levels of amyloid Aβ_1−42_, in line with previous studies [74, 75]. However, we recognize that such relation was much stronger and statistically stable for tau proteins only, being less consistent and very imprecise for amyloid Aβ_1−42_. Such difference may suggest a stronger effect of LAN in the progression of neurodegeneration process specifically through alteration of tau proteins as indicated.

Our results are supported by some laboratory studies suggesting that LAN may increase neurodegeneration. In particular, dim light exposure during night hours induced disruption of circadian rhythm and sleep thus promoting accumulation of tau protein in the brains of tauopathy/AD Drosophila fly model [43]. Similarly, light exposure increases tau cleavage and neurodegeneration in the Drosophila spaghetti fly model [44]. Despite most of the attention has been devoted to circadian rhythm alteration and clock genes expression [32, 47, 50], the exact mechanisms of the effects of LAN exposure are not fully understood and several studies have indicated that they are not limited to an altered clock daytime perception. As a matter of fact, altered expression of genes related to oxidative stress (e.g., altered expression of heat shock proteins, lactate dehydrogenase, or lipid peroxidation) have been described [44, 48, 76], or altered nighttime activity and behavior as well as metabolism and hormone secretion [77–80]. Finally, some animal studies suggested that LAN negatively impacts memory and cognitive performances [76, 81–84], specifically affecting brain regions linked to learning and memory like hippocampus [76, 85]. In particular, LAN exposure may alter vascular structure and function of the hippocampus [86]. Nonetheless, other independent mechanisms may affect such relation. For instance, sleep disorders and deprivation have been suggested to dysregulate amyloid-beta clearance/deposition [87–90], thus affecting neurodegeneration independently from LAN exposure. With regards to tau protein, it is also known to be released into the extracellular space during periods of heightened excitatory neuronal activity [91]. Hence, we speculate that a prolonged elevation in neuronal activity due to LAN might enhance tau propagation and contribute to pathological processes.

An important limit of our analysis is the limited sample size affecting the statistical precision of the risk estimates and the interpretation of the results, indicating caution in the generalization to other population and the need to further assess such relation in larger investigations. In addition, despite we included major confounders and adjusted by other relevant environmental factors like smoking, traffic-related air particulate matter, some chronic disease, and partially also genetic factors, the observational study design does not entirely rule out the occurrence of residual confounding due to other factors not considered in the analysis. In particular, we did not assess greenness exposure, a factor recently associated with a non-linear U-shaped pattern with both dementia risk [24] and neuropsychiatric symptoms in subjects with dementia [27]. Although, several indicators can be used, such as living close to green spaces or satellite-based assessments like Normalized Difference Vegetation Index (NDVI) [92], several factors (especially modality and resolution) may affect their use and application in epidemiologic studies [93] and specifically hampered its assessment to this study population. Due to the study design, we could not retrieve information of indoor artificial LAN exposure as well as night shift working, although both indoor and outdoor lighting showed to affect each other and have similar intensity and spectral composition [94]. Similarly, exposure assessment using VIIRS data hampered the evaluation of possible difference of specific spectra, specifically a higher detrimental effect of blue light [47, 48].

In addition, we cannot entirely exclude risk of misclassification of exposure using residential data although we did not contact study subjects and we obtained median LAN exposure (26.4 nW/cm^2^/sr) almost identical with the previous investigation in the same Northern Italy province (25.8 nW/cm^2^/sr) [52], although lower than the Chinese study (44.4 nW/cm^2^/sr) [51]. Despite such difference partially limits the comparison of our results with other studies, the implementation of several categorical analyses and especially non-linear splines over the entire range of exposure levels should substantially improve risk characterization and ease the use of these findings in meta-analyses.

Some strengths of the present study should be highlighted. For exposure assessment, we used a valid method based on satellite data within a geographic information system, characterized by the highest available spatial resolution using of VIIRS imagery data [95], implementing both correlation and sensitivity analysis across different available datasets. In addition, to the best of our knowledge, this is the first prospective study assessing the relation between outdoor artificial LAN and risk of dementia conversion. The use of LAN levels from VIIRS data from either the follow-up period (2014–2022 9-year average) or at the begin at follow-up (2014), should rule out risk of misclassification and reverse causation due to change of exposure after dementia diagnosis [96].

## Conclusions

Our findings suggest that outdoor artificial LAN may increase dementia conversion, especially above 30 nW/cm^2^/sr, although the limited sample size suggests caution in the interpretation of the results, and the need to further evaluate such relation in larger studies.

## Electronic supplementary material

Below is the link to the electronic supplementary material.

Supplementary Material 1

## Data Availability

Data used are confidential due to their sensitive nature. Raw individual data of study participants must remain confidential and cannot be shared.

## Abbreviations

amyloid Aβ_1−42_: Amyloid Abeta_1 − 42_
AD: Alzheimer’s dementia
APOE4: Apolipoprotein E ε4 genotype
CI: Confidence interval
CNS: Central nervous system
COPD: Chronic obstructive pulmonary disease
CSF: Cerebrospinal fluid
FTD: Frontotemporal dementia
HR: Hazard ratio
IQR: Interquartile range
LAN: Light at night
LBD: Lewy body dementia
MCI: Mild cognitive impairment
p-tau181: Phosphorylated tau
PM_10_: <10 μm particulate matter
t-tau: Total tau
