# Supplementary material for: Light at night exposure and risk of dementia conversion from mild cognitive impairment in a Northern Italy population
Source: Int J Health Geogr. 2024 Nov 23;23:25. doi: 10.1186/s12942-024-00384-5 (PMC11585219; doi:10.1186/s12942-024-00384-5)
Supplement: Supplementary file 1 — Supplementary Material 1 [file 12942_2024_384_MOESM1_ESM.docx]

**SUPPLEMENTARY MATERIAL**

**Supplementary Table S1.** Baseline characteristics of study participants with mild cognitive impairment according to diagnosis at the end of follow-up for other dementia forms: frontotemporal dementia (FTD), Lewy body dementia (LBD), and vascular dementia (VaD).

|  | FTD | | LBD | | VaD | |
| --- | --- | --- | --- | --- | --- | --- |
|  | **N** | **%** | **N** | **%** | **N** | **%** |
| All subjects | 4 | 100 | 2 | 100 | 2 | 100 |
| Sex |  |  |  |  |  |  |
| Males | 2 | 50.0 | 1 | 50.0 | 2 | 100.0 |
| Females | 2 | 50.0 | 1 | 50.0 | 0 | 0.0 |
| Age at first diagnosis |  |  |  |  |  |  |
| <65 years | 4 | 100.0 | 0 | 0.0 | 0 | 0.0 |
| ≥65 years | 0 | 0.0 | 2 | 100.0 | 2 | 100.0 |
| Educational attainment |  |  |  |  |  |  |
| <8 years | 0 | 0.0 | 2 | 100.0 | 0 | 0.0 |
| 8-12 years | 0 | 0.0 | 0 | 0.0 | 2 | 100.0 |
| ≥12 years | 4 | 100.0 | 0 | 0.0 | 0 | 0.0 |
| Smoking habits |  |  |  |  |  |  |
| Non-smokers | 3 | 75.0 | 2 | 100.0 | 2 | 100.0 |
| Smokers | 1 | 25.0 | 0 | 0.0 | 0 | 0.0 |
| COPD |  |  |  |  |  |  |
| No | 3 | 75.0 | 2 | 100.0 | 2 | 100.0 |
| Yes | 1 | 25.0 | 0 | 0.0 | 0 | 0.0 |
| Diabetes |  |  |  |  |  |  |
| No | 4 | 100.0 | 2 | 100.0 | 2 | 100.0 |
| Yes | 0 | 0.0 | 0 | 0.0 | 0 | 0.0 |
| APOE4 positivity |  |  |  |  |  |  |
| Negative | 3 | 75.0 | 1 | 50.0 | 1 | 50.0 |
| Positive | 0 | 0.0 | 1 | 50.0 | 1 | 50.0 |
| Missing | 1 | 25.0 | 0 | 0.0 | 0 | 0.0 |
| Follow-up (months)^a^ | 43 | - | 38 | - | 98 | - |

^a^Median value reported only. APOE4, apolipoprotein E ε4 genotype; COPD, chronic obstructive pulmonary disease.

**Supplementary Table S2.** Distribution of Visible Infrared Imaging Radiometer Suite (VIIRS) data from 2014 to 2022 and 2014-2022 average divided by participants’ characteristics. Median and interquartile range values in nW/cm^2^/sr.

|  | 2014-2022 | VIIRS 2014 | VIIRS 2015 | VIIRS 2016 | VIIRS 2017 | VIIRS 2018 | VIIRS 2019 | VIIRS 2020 | VIIRS 2021 | VIIRS 2022 |
| --- | --- | --- | --- | --- | --- | --- | --- | --- | --- | --- |
| All subjects (n=53) | 26.2  (14.9-33.8) | 27.9  (15.7-32.4) | 27.6  (15.9-35.8) | 26.7  (16.4-32.7) | 27.3  (14.9-34.3) | 25.2  (14.0-33.6) | 26.3  (15.0-35.0) | 25.0  (14.7-31.5) | 25.7  (16.3-31.4) | 26.4  (15.1-35.8) |
| Sex |  |  |  |  |  |  |  |  |  |  |
| Males  (n=28) | 26.2  (14.1-34.1) | 26.5  (13.9-31.6) | 27.1  (13.6-35.8) | 27.6  (14.6-36.4) | 26.2  (13.6-35.0) | 25.2  (13.4-34.3) | 26.0  (13.0-35.1) | 23.6  (13.2-31.9) | 24.2  (13.6-31.1) | 25.2  (14.1-32.0) |
| Females  (n=25) | 26.4  (16.9-32.8) | 28.0  (17.3-35.1) | 27.6  (15.9-35.8) | 26.7  (16.4-34.7) | 29.1  (15.7-34.3) | 25.2  (15.8-32.7) | 27.1  (17.1-35.0) | 27.3  (17.6-31.2) | 27.9  (19.3-32.1) | 27.2  (19.3-35.8) |
| Age |  |  |  |  |  |  |  |  |  |  |
| <65 years  (n=22) | 25.6  (14.5-32.8) | 24.3  (15.0-32.4) | 24.6  (13.7-33.6) | 25.2  (13.6-34.7) | 25.4  (14.5-34.3) | 24.1  (13.8-32.7) | 25.2  (15.0-35.7) | 24.2  (14.7-31.2) | 26.1  (16.3-29.9) | 26.0  (15.1-32.2) |
| ≥65 yeas  (n=31) | 26.3  (15.7-34.3) | 28.1  (17.2-35.3) | 28.7  (17.8-36.4) | 29.9  (17.1-36.4) | 29.1  (16.535.1) | 27.3  (14.4-33.7) | 29.5  (14.7-34-7) | 26.3  (14.5-32.4) | 25.7  (14.2-32.1) | 27.2  (15.1-34.2) |
| Education |  |  |  |  |  |  |  |  |  |  |
| <8 years  (n=18) | 31.4  (23.1-36.0) | 29.7  (20.7-35.3) | 31.8  (20.0-37.6) | 31.3  (21.9-38.1) | 30.0  (23.2-38.3) | 30.6  (21.3-38.0) | 29.9  (21.9-35.7) | 29.1  (21.4-32.4) | 28.7  (24.2-32.9) | 31.6  (25.6-35.8) |
| 8-12 years  (n=15) | 26.1  (14.4-33.0) | 28.4  (13.2-35.1) | 28.7  (12.7-35.8) | 32.2  (12.7-34.6) | 29.9  (14.5-35.1) | 25.2  (14.0-34.9) | 26.3  (14.7-35.7) | 26.3  (14.5-32.6) | 24.0  (14.2-30.6) | 24.9  (15.1-33.2) |
| ≥12 years  (n=20) | 24.7  (11.2-31.7) | 22.7  (11.7-32.4) | 23.0  (11.7-33.2) | 25.1  (11.7-33.5) | 24.9  (11.4-31.2) | 24.2  (10.9-31.2) | 25.1  (10.0-31.8) | 22.8  (10.1-29.3) | 23.1  (10.6-30.2) | 24.8  (10.9-30.4) |
| Smoking habits |  |  |  |  |  |  |  |  |  |  |
| Non-smokers (n=46) | 26.2  (13.6-33.8) | 26.5  (15.0-32.4) | 26.5  (13.7-35.8) | 26.3  (13.6-34.7) | 26.7  (12.6-35.1) | 25.2  (12.9-33,7) | 26.2  (11.2-35.5) | 25.0  (11.8-32.4) | 25.4  (12.9-30.8) | 26.3  (13.9-33.8) |
| Smokers  (n=7) | 26.2  (25.4-34.4) | 28.5  (23.5-37.0) | 28.7  (23.9-41.0) | 30.8  (25.4-38.7) | 29.1  (25.5-31.7) | 30.0  (24.0-32.3) | 29.5  (24.6-34.3) | 27.3  (22.8-31.3) | 27.9  (23.9-32.9) | 28.6  (24.6-35.8) |
| COPD |  |  |  |  |  |  |  |  |  |  |
| No  (n=51) | 26.4  (14.5-34.3) | 27.9  (15.7-35.1) | 27.6  (15.5-36.4) | 26.7  (14.3-36.0) | 28.5  (14.5-35.0) | 25.9  (13.8-33.7) | 26.3  (14.7-35.5) | 25.0  (14.5-32.4) | 25.7  (14.2-31.8) | 26.4  (15.1-34.3) |
| Yes  (n=2) | 25.7  (25.4-26.1) | 26.0  (23.5-28.4) | 26.3  (23.9-28.7) | 26.6  (21.1-32.2) | 26.4  (25.5-27.3) | 24.1  (24.0-24.3) | 25.4  (24.6-26.2) | 25.0  (22.8-27.3) | 25.9  (23.9-27.9) | 25.8  (22.9-28.6) |
| Diabetes |  |  |  |  |  |  |  |  |  |  |
| No  (n=51) | 26.2  (14.9-34.3) | 27.9  (15.7-35.1) | 27.6  (15.9-36.4) | 26.7  (16.4-36.0) | 27.3  (14.9-34.9) | 25.2  (13.8-33.6) | 26.2  (14.7-35.0) | 25.0  (14.5-32.4) | 25.7  (14.2-31.8) | 26.4  (15.1-34.3) |
| Yes  (n=2) | 23.7  (14.5-32.8) | 21.9  (12.1-31.7) | 22.3  (11.2-33.6) | 23.7  (12.7-34.6) | 26.4  (14.5-38.3) | 26.0  (14.0-38.0) | 25.7  (15.6-35.7) | 22.0  (15.1-28.2) | 21.7  (16.3-27.1) | 23.2  (18.9-27.6) |
| APOE4 genotype |  |  |  |  |  |  |  |  |  |  |
| Negative  (n=21) | 25.4  (13.6-34.4) | 23.5  (13.2-31.6) | 23.9  (12.7-33.5) | 24.4  (12.7-34.7) | 24.6  (12.6-33.1) | 24.3  (12.9-32.7) | 25.8  (11.2-34.7) | 23.8  (11.8-33.7) | 24.2  (12.6-31.4) | 24.9  (13.0-36.0) |
| Positive  (n=17) | 31.0  (16.9-33.0) | 28.8  (17.3-35.3) | 31.2  (15.9-35.8) | 32.2  (16.4-36.0) | 29.1  (15.7-35.1) | 30.0  (15.8-34.9) | 29.5  (17.1-35.5) | 29.5  (17.6-31.5) | 27.4  (19.3-32.1) | 31.5  (20.2-34.3) |
| Missing  (n=15) | 25.7  (23.1-32.8) | 25.0  (21.7-35.1) | 25.3  (21.3-36.4) | 25.9  (22.1-34.6) | 27.2  (23.2-38.3) | 25.9  (21.3-34.3) | 26.2  (21.9-35.7) | 24.9  (21.4-28.8) | 25.1  (21.7-29.9) | 26.2  (19.3-27.6) |

Abbreviations: APOE4, apolipoprotein E ε4 genotype; COPD, chronic obstructive pulmonary disease.

**Supplementary Table S3.** Correlation matrix across Visible Infrared Imaging Radiometer Suite (VIIRS) data from 2014 to 2022 and 2014-2022 average.

|  | **VIIRS 2022** | **VIIRS 2021** | **VIIRS 2020** | **VIIRS 2019** | **VIIRS 2018** | **VIIRS 2017** | **VIIRS 2016** | **VIIRS 2015** | **VIIRS 2014** |
| --- | --- | --- | --- | --- | --- | --- | --- | --- | --- |
| **VIIRS 2022** | 1 |  |  |  |  |  |  |  |  |
| **VIIRS 2021** | 0.986 | 1 |  |  |  |  |  |  |  |
| **VIIRS 2020** | 0.971 | 0.983 | 1 |  |  |  |  |  |  |
| **VIIRS 2019** | 0.926 | 0.940 | 0.974 | 1 |  |  |  |  |  |
| **VIIRS 2018** | 0.925 | 0.935 | 0.964 | 0.992 | 1 |  |  |  |  |
| **VIIRS 2017** | 0.915 | 0.929 | 0.955 | 0.980 | 0.987 | 1 |  |  |  |
| **VIIRS 2016** | 0.921 | 0.928 | 0.944 | 0.953 | 0.964 | 0.975 | 1 |  |  |
| **VIIRS 2015** | 0.909 | 0.914 | 0.935 | 0.950 | 0.956 | 0.967 | 0.991 | 1 |  |
| **VIIRS 2014** | 0.934 | 0.944 | 0.951 | 0.940 | 0.947 | 0.963 | 0.985 | 0.985 | 1 |
| **VIIRS 2014-2022 average** | 0.952 | 0.963 | 0.980 | 0.984 | 0.987 | 0.988 | 0.986 | 0.981 | 0.983 |

**Supplementary Table S4.** Risk of dementia according to increasing exposure to outdoor artificial light at night (LAN) using 9-year average 2014-2022 period of Visible Infrared Imaging Radiometer Suite (VIIRS) data, using both linear increase and categories based on median (50th), tertiles, and fixed cutoffs of nighttime luminance exposure (LAN) in nW/cm^2^/sr, in a multivariable analysis stratified by sex and adjusted by age, and educational attainment, further adjusted by particulate matter concentrations. Analysis considering as outcome any dementia, and Alzheimer’s dementia only (with exclusion of other dementia cases ab initio). HR: hazard ratio, CI: confidence interval.

| **2014-2022** |  |  |  | **Any dementia** |  |  |  |  | **AD** |  |
| --- | --- | --- | --- | --- | --- | --- | --- | --- | --- | --- |
| **LAN (nW/cm^2^/sr)** | **50^th^** | **N**  **C+/C-** | **OR** | **(95% CI)** | **P value** | **50^th^** | **N**  **C+/C-** | **OR** | **(95% CI)** | **P value** |
| Linear trend (1-unit increase) | - |  | 1.04 | (1.01-1.08) | 0.020 | - |  | 1.04 | (1.00-1.08) | 0.045 |
| Linear trend (10-unit increase) | - |  | 1.53 | (1.07-2.18) | 0.020 | - |  | 1.51 | (1.01-2.25) | 0.045 |
| **LAN-Median** |  |  |  |  |  |  |  |  |  |  |
| Below the median | 14.7 | 15/11 | 1.00 | - |  | 14.1 | 11/11 | 1.00 | - |  |
| Above or equal the median | 33.8 | 19/8 | 1.81 | (0.82-3.99) | 0.141 | 33.8 | 15/8 | 1.91 | (0.77-4.78) | 0.165 |
| **LAN-Tertiles** |  |  |  |  |  |  |  |  |  |  |
| 1^st^ tertile | 9.7 | 8/9 | 1.00 | - |  | 9.7 | 6/9 | 1.00 | - |  |
| 2^nd^ tertile | 26.2 | 13/5 | 2.94 | (1.07-8.07) | 0.036 | 26.3 | 9/5 | 2.66 | (0.82-8.64) | 0.105 |
| 3^rd^ tertile | 36.4 | 13/5 | 4.42 | (1.44-13.59) | 0.009 | 36.4 | 11/5 | 4.89 | (1.33-17.97) | 0.017 |
| **LAN-Fixed cut-offs** |  |  |  |  |  |  |  |  |  |  |
| <15 | 9.2 | 7/7 | 1.00 | - |  | 8.8 | 6/7 | 1.00 | - |  |
| ≥15; < 30 | 25.4 | 10/7 | 2.29 | (0.77-6.83) | 0.136 | 25.7 | 6/7 | 1.61 | (0.47-5.7) | 0.450 |
| ≥ 30 | 35.0 | 17/5 | 4.32 | (1.39-13.38) | 0.011 | 34.4 | 14/5 | 3.46 | (1.02-11.75) | 0.046 |

**Supplementary Table S5.** Risk of dementia according to increasing exposure to outdoor artificial light at night (LAN) using 9-year average 2014-2022 period of Visible Infrared Imaging Radiometer Suite (VIIRS) data, using both linear increase and categories based on median (50th), tertiles, and fixed cutoffs of nighttime luminance exposure (LAN) in nW/cm^2^/sr, in a multivariable analysis stratified by sex and adjusted by age, and educational attainment, and further adjusted by smoking status. Analysis considering as outcome any dementia, and Alzheimer’s dementia only (with exclusion of other dementia cases ab initio). HR: hazard ratio, CI: confidence interval.

| **2014-2022** |  |  |  | **Any dementia** |  |  |  |  | **AD** |  |
| --- | --- | --- | --- | --- | --- | --- | --- | --- | --- | --- |
| **LAN (nW/cm^2^/sr)** | **50^th^** | **N**  **C+/C-** | **OR** | **(95% CI)** | **P value** | **50^th^** | **N**  **C+/C-** | **OR** | **(95% CI)** | **P value** |
| Linear trend (1-unit increase) | - |  | 1.04 | (1.00-1.07) | 0.024 | - |  | 1.01 | (1.00-1.07) | 0.066 |
| Linear trend (10-unit increase) | - |  | 1.45 | (1.05-1.99) | 0.024 | - |  | 1.39 | (0.98-1.98) | 0.066 |
| **LAN-Median** |  |  |  |  |  |  |  |  |  |  |
| Below the median | 14.7 | 15/11 | 1.00 | - |  | 14.1 | 11/11 | 1.00 | - |  |
| Above or equal the median | 33.8 | 19/8 | 1.83 | (0.83-4.01) | 0.132 | 33.8 | 15/8 | 1.83 | (0.76-4.38) | 0.177 |
| **LAN-Tertiles** |  |  |  |  |  |  |  |  |  |  |
| 1^st^ tertile | 9.7 | 8/9 | 1.00 | - |  | 9.7 | 6/9 | 1.00 | - |  |
| 2^nd^ tertile | 26.2 | 13/5 | 2.65 | (0.99-7.07) | 0.052 | 26.3 | 9/5 | 2.16 | (0.67-6.90) | 0.195 |
| 3^rd^ tertile | 36.4 | 13/5 | 3.74 | (1.35-10.42) | 0.011 | 36.4 | 11/5 | 3.62 | (1.15-11.38) | 0.028 |
| **LAN-Fixed cut-offs** |  |  |  |  |  |  |  |  |  |  |
| <15 | 9.2 | 7/7 | 1.00 | - |  | 8.8 | 6/7 | 1.00 | - |  |
| ≥15; < 30 | 25.4 | 10/7 | 2.09 | (0.73-5.93) | 0.167 | 25.7 | 6/7 | 1.45 | (0.44-4.75) | 0.539 |
| ≥ 30 | 35.0 | 17/5 | 3.87 | (1.33-11.22) | 0.013 | 34.4 | 14/5 | 3.03 | (0.97-9.43) | 0.056 |

**Supplementary Table S6.** Risk of dementia according to increasing exposure to outdoor artificial light at night (LAN) using 9-year average 2014-2022 period of Visible Infrared Imaging Radiometer Suite (VIIRS) data, using both linear increase and categories based on median (50th), tertiles, and fixed cutoffs of nighttime luminance exposure (LAN) in nW/cm^2^/sr, in a multivariable analysis stratified by sex and adjusted by age, and educational attainment, and further adjusted by chronic obstructive pulmonary disease. Analysis considering as outcome any dementia, and Alzheimer’s dementia only (with exclusion of other dementia cases ab initio). HR: hazard ratio, CI: confidence interval.

| **2014-2022** |  |  |  | **Any dementia** |  |  |  |  | **AD** |  |
| --- | --- | --- | --- | --- | --- | --- | --- | --- | --- | --- |
| **LAN (nW/cm^2^/sr)** | **50^th^** | **N**  **C+/C-** | **OR** | **(95% CI)** | **P value** | **50^th^** | **N**  **C+/C-** | **OR** | **(95% CI)** | **P value** |
| Linear trend (1-unit increase) | - |  | 1.04 | (1.00-1.07) | 0.020 | - |  | 1.03 | (1.00-1.07) | 0.056 |
| Linear trend (10-unit increase) | - |  | 1.46 | (1.06-2.01) | 0.020 | - |  | 1.40 | (0.99-1.99) | 0.056 |
| **LAN-Median** |  |  |  |  |  |  |  |  |  |  |
| Below the median | 14.7 | 15/11 | 1.00 | - |  | 14.1 | 11/11 | 1.00 | - |  |
| Above or equal the median | 33.8 | 19/8 | 2.10 | (0.96-4.57) | 0.062 | 33.8 | 15/8 | 2.10 | (0.88-5.01) | 0.093 |
| **LAN-Tertiles** |  |  |  |  |  |  |  |  |  |  |
| 1^st^ tertile | 9.7 | 8/9 | 1.00 | - |  | 9.7 | 6/9 | 1.00 | - |  |
| 2^nd^ tertile | 26.2 | 13/5 | 2.29 | (0.87-6.03) | 0.095 | 26.3 | 9/5 | 1.86 | (0.60-5.76) | 0.280 |
| 3^rd^ tertile | 36.4 | 13/5 | 3.67 | (1.35-9.95) | 0.011 | 36.4 | 11/5 | 3.68 | (1.22-11.15) | 0.021 |
| **LAN-Fixed cut-offs** |  |  |  |  |  |  |  |  |  |  |
| <15 | 9.2 | 7/7 | 1.00 | - |  | 8.8 | 6/7 | 1.00 | - |  |
| ≥15; < 30 | 25.4 | 10/7 | 1.73 | (0.59-5.06) | 0.318 | 25.7 | 6/7 | 1.16 | (0.34-3.96) | 0.808 |
| ≥ 30 | 35.0 | 17/5 | 3.65 | (1.34-9.95) | 0.012 | 34.4 | 14/5 | 2.87 | (1.01-8.15) | 0.048 |

**Supplementary Table S7.** Risk of dementia according to increasing exposure to outdoor artificial light at night (LAN) using 9-year average 2014-2022 period of Visible Infrared Imaging Radiometer Suite (VIIRS) data, using both linear increase and categories based on median (50th), tertiles, and fixed cutoffs of nighttime luminance exposure (LAN) in nW/cm^2^/sr, in a multivariable analysis stratified by age and adjusted by age, and educational attainment, and further adjusted by diabetes. Analysis considering as outcome any dementia, and Alzheimer’s dementia only (with exclusion of other dementia cases ab initio). HR: hazard ratio, CI: confidence interval.

| **2014-2022** |  |  |  | **Any dementia** |  |  |  |  | **AD** |  |
| --- | --- | --- | --- | --- | --- | --- | --- | --- | --- | --- |
| **LAN (nW/cm^2^/sr)** | **50^th^** | **N**  **C+/C-** | **OR** | **(95% CI)** | **P value** | **50^th^** | **N**  **C+/C-** | **OR** | **(95% CI)** | **P value** |
| Linear trend (1-unit increase) | - |  | 1.04 | (1.01-1.07) | 0.021 | - |  | 1.03 | (1.00-1.07) | 0.052 |
| Linear trend (10-unit increase) | - |  | 1.44 | (1.06-1.96) | 0.021 | - |  | 1.40 | (1.00-1.97) | 0.052 |
| **LAN-Median** |  |  |  |  |  |  |  |  |  |  |
| Below the median | 14.7 | 15/11 | 1.00 | - |  | 14.1 | 11/11 | 1.00 | - |  |
| Above or equal the median | 33.8 | 19/8 | 1.86 | (0.88-3.90) | 0.103 | 33.8 | 15/8 | 1.91 | (0.82-4.45) | 0.133 |
| **LAN-Tertiles** |  |  |  |  |  |  |  |  |  |  |
| 1^st^ tertile | 9.7 | 8/9 | 1.00 | - |  | 9.7 | 6/9 | 1.00 | - |  |
| 2^nd^ tertile | 26.2 | 13/5 | 2.50 | (0.98-6.43) | 0.056 | 26.3 | 9/5 | 2.14 | (0.71-6.46) | 0.175 |
| 3^rd^ tertile | 36.4 | 13/5 | 3.71 | (1.37-10.06) | 0.010 | 36.4 | 11/5 | 3.71 | (1.23-11.18) | 0.020 |
| **LAN-Fixed cut-offs** |  |  |  |  |  |  |  |  |  |  |
| <15 | 9.2 | 7/7 | 1.00 | - |  | 8.8 | 6/7 | 1.00 | - |  |
| ≥15; < 30 | 25.4 | 10/7 | 1.97 | (0.70-5.51) | 0.198 | 25.7 | 6/7 | 1.40 | (0.44-4.52) | 0.570 |
| ≥ 30 | 35.0 | 17/5 | 3.59 | (1.33-9.72) | 0.012 | 34.4 | 14/5 | 2.90 | (1.02-8.17) | 0.045 |

**Supplementary Table S8.** Risk of dementia according to increasing exposure to outdoor artificial light at night (LAN) using 9-year average 2014-2022 period of Visible Infrared Imaging Radiometer Suite (VIIRS) data, using both linear increase and categories based on median (50th), tertiles, and fixed cutoffs of nighttime luminance exposure (LAN) in nW/cm^2^/sr, in a multivariable analysis stratified by sex and adjusted by age, and educational attainment, and further adjusted by apolipoprotein E ε4 genotype status. Analysis considering as outcome any dementia, and Alzheimer’s dementia only (with exclusion of other dementia cases ab initio). HR: hazard ratio, CI: confidence interval.

| **2014-2022** |  |  |  | **Any dementia** |  |  |  |  | **AD** |  |
| --- | --- | --- | --- | --- | --- | --- | --- | --- | --- | --- |
| **LAN (nW/cm^2^/sr)** | **50^th^** | **N**  **C+/C-** | **OR** | **(95% CI)** | **P value** | **50^th^** | **N**  **C+/C-** | **OR** | **(95% CI)** | **P value** |
| Linear trend (1-unit increase) | - |  | 1.03 | (0.99-1.06) | 0.168 | - |  | 1.02 | (0.98-1.06) | 0.433 |
| Linear trend (10-unit increase) | - |  | 1.29 | (0.90-1.87) | 0.168 | - |  | 1.18 | (0.78-1.78) | 0.433 |
| **LAN-Median** |  |  |  |  |  |  |  |  |  |  |
| Below the median | 13.6 | 15/6 | 1.00 | - |  | 12.2 | 11/6 | 1.00 | - |  |
| Above or equal the median | 33.8 | 10/7 | 1.54 | (0.62-3.80) | 0.353 | 33.8 | 7/7 | 1.59 | (0.56-4.48) | 0.380 |
| **LAN-Tertiles** |  |  |  |  |  |  |  |  |  |  |
| 1^st^ tertile | 10.2 | 7/7 | 1.00 | - |  | 10.2 | 5/7 | 1.00 | - |  |
| 2^nd^ tertile | 26.5 | 9/2 | 2.20 | (0.72-6.73) | 0.168 | 26.9 | 6/2 | 1.46 | (0.38-5.58) | 0.577 |
| 3^rd^ tertile | 36.74 | 9/4 | 2.81 | (0.92-8.62) | 0.070 | 36.7 | 7/4 | 2.51 | (0.70-8.94) | 0.156 |
| **LAN-Fixed cut-offs** |  |  |  |  |  |  |  |  |  |  |
| <15 | 9.7 | 6/5 | 1.00 | - |  | 9.2 | 5/5 | 1.00 | - |  |
| ≥15; < 30 | 25.8 | 6/4 | 1.64 | (0.49-5.48) | 0.420 | 26.2 | 3/4 | 0.88 | (0.20-3.87) | 0.866 |
| ≥ 30 | 35.5 | 13/4 | 2.41 | (0.79-7.33) | 0.121 | 35.2 | 10/4 | 1.60 | (0.79-6.26) | 0.438 |

**Supplementary Table S9.** Risk of dementia according to increasing exposure to outdoor artificial light at night (LAN) using 9-year average 2014-2022 period of Visible Infrared Imaging Radiometer Suite (VIIRS) data, using both linear increase and categories based on median (50th), tertiles, and fixed cutoffs of nighttime luminance exposure (LAN) in nW/cm^2^/sr, in a multivariable analysis stratified by sex and adjusted by age, and educational attainment, and restricted to non-smokers. Analysis considering as outcome any dementia, and Alzheimer’s dementia only (with exclusion of other dementia cases ab initio). HR: hazard ratio, CI: confidence interval.

| **2014-2022** |  |  |  | **Any dementia** |  |  |  |  | **AD** |  |
| --- | --- | --- | --- | --- | --- | --- | --- | --- | --- | --- |
| **LAN (nW/cm^2^/sr)** | **50^th^** | **N**  **C+/C-** | **OR** | **(95% CI)** | **P value** | **50^th^** | **N**  **C+/C-** | **OR** | **(95% CI)** | **P value** |
| Linear trend (1-unit increase) | - |  | 1.04 | (1.01-1.08) | 0.016 | - |  | 1.04 | (1.00-1.08) | 0.043 |
| Linear trend (10-unit increase) | - |  | 1.51 | (1.08-2.11) | 0.016 | - |  | 1.47 | (1.01-2.13) | 0.043 |
| **LAN-Median** |  |  |  |  |  |  |  |  |  |  |
| Below the median | 13.6 | 11/7 | 1.00 | - |  | 13.6 | 8/11 | 1.00 | - |  |
| Above or equal the median | 33.8 | 16/7 | 2.77 | (1.15-6.66) | 0.023 | 33.4 | 13/7 | 3.02 | (1.10-8.29) | 0.032 |
| **LAN-Tertiles** |  |  |  |  |  |  |  |  |  |  |
| 1^st^ tertile | 9.7 | 8/7 | 1.00 | - |  | 8.8 | 6/7 | 1.00 | - |  |
| 2^nd^ tertile | 26.0 | 8/7 | 1.68 | (0.60-4.71) | 0.328 | 26.1 | 5/7 | 1.17 | (0.34-4.04) | 0.809 |
| 3^rd^ tertile | 36.4 | 12/4 | 3.80 | (1.30-11.11) | 0.015 | 36.4 | 10/4 | 3.61 | (1.11-11.81) | 0.034 |
| **LAN-Fixed cut-offs** |  |  |  |  |  |  |  |  |  |  |
| <15 | 9.2 | 7/7 | 1.00 | - |  | 8.8 | 6/7 | 1.00 | - |  |
| ≥15; < 30 | 25.4 | 6/7 | 1.51 | (0.48-4.77) | 0.487 | 25.6 | 3/7 | 0.81 | (0.19-3.42) | 0.776 |
| ≥ 30 | 35.5 | 15/4 | 5.08 | (1.63-15.89) | 0.005 | 35.1 | 12/4 | 3.78 | (1.16-12.34) | 0.027 |

**Supplementary Table S10.** Risk of dementia according to increasing exposure to outdoor artificial light at night (LAN) using 9-year average 2014-2022 period of Visible Infrared Imaging Radiometer Suite (VIIRS) data, using both linear increase and categories based on median (50th), tertiles, and fixed cutoffs of nighttime luminance exposure (LAN) in nW/cm^2^/sr, in a multivariable analysis stratified by sex and adjusted by age, and educational attainment, Analysis excluding subjects with diagnosis within 12 months. Analysis considering as outcome any dementia, and Alzheimer’s dementia only (with exclusion of other dementia cases ab initio). HR: hazard ratio, CI: confidence interval.

| **2014-2022** |  |  |  | **Any dementia** |  |  |  |  | **AD** |  |
| --- | --- | --- | --- | --- | --- | --- | --- | --- | --- | --- |
| **LAN (nW/cm^2^/sr)** | **50^th^** | **N**  **C+/C-** | **OR** | **(95% CI)** | **P value** | **50^th^** | **N**  **C+/C-** | **OR** | **(95% CI)** | **P value** |
| Linear trend (1-unit increase) | - |  | 1.04 | (1.01-1.07) | 0.023 | - |  | 1.03 | (1.00-1.07) | 0.056 |
| Linear trend (10-unit increase) | - |  | 1.44 | (1.05-1.98) | 0.023 | - |  | 1.40 | (0.99-1.99) | 0.056 |
| **LAN-Median** |  |  |  |  |  |  |  |  |  |  |
| Below the median | 14.5 | 14/11 | 1.00 | - |  | 13.6 | 10/11 | 1.00 | - |  |
| Above or equal the median | 34.0 | 19/7 | 1.99 | (0.93-4.23) | 0.076 | 34.0 | 15/7 | 2.10 | (0.88-5.01) | 0.093 |
| **LAN-Tertiles** |  |  |  |  |  |  |  |  |  |  |
| 1^st^ tertile | 9.7 | 8/9 | 1.00 | - |  | 9.2 | 6/8 | 1.00 | - |  |
| 2^nd^ tertile | 26.2 | 12/5 | 2.33 | (0.90-6.08) | 0.083 | 26.3 | 8/6 | 1.72 | (0.57-5.18) | 0.337 |
| 3^rd^ tertile | 36.7 | 13/4 | 3.66 | (1.35-9.93) | 0.011 | 36.7 | 11/4 | 3.54 | (1.17-10.66) | 0.025 |
| **LAN-Fixed cut-offs** |  |  |  |  |  |  |  |  |  |  |
| <15 | 9.2 | 7/7 | 1.00 | - |  | 8.8 | 6/7 | 1.00 | - |  |
| ≥15; < 30 | 25.4 | 9/7 | 1.80 | (0.63-5.15) | 0.272 | 25.6 | 5/7 | 1.16 | (0.34-3.96) | 0.808 |
| ≥ 30 | 35.5 | 17/4 | 3.59 | (1.32-9.80) | 0.012 | 35.2 | 14/4 | 2.87 | (1.01-8.15) | 0.048 |

**Supplementary Table S11.** Risk of dementia according to increasing exposure to outdoor artificial light at night (LAN) using mean annual 2014 Visible Infrared Imaging Radiometer Suite (VIIRS) data, using both linear increase and categories based on median (50th), tertiles, and fixed cutoffs of nighttime luminance exposure (LAN) in nW/cm^2^/sr, in a multivariable analysis stratified by sex and adjusted by age, and educational attainment. Analysis considering as outcome any dementia, and Alzheimer’s dementia only (with exclusion of other dementia cases ab initio). HR: hazard ratio, CI: confidence interval.

| **2014** |  |  |  | **Any dementia** |  |  |  |  | **AD** |  |
| --- | --- | --- | --- | --- | --- | --- | --- | --- | --- | --- |
| **LAN (nW/cm^2^/sr)** | **50^th^** | **N**  **C+/C-** | **OR** | **(95% CI)** | **P value** | **50^th^** | **N**  **C+/C-** | **OR** | **(95% CI)** | **P value** |
| Linear trend (1-unit increase) | - |  | 1.03 | (1.00-1.07) | 0.038 | - |  | 1.03 | (1.00-1.07) | 0.079 |
| Linear trend (10-unit increase) | - |  | 1.40 | (1.02-1.92) | 0.038 | - |  | 1.36 | (0.96-1.92) | 0.079 |
| **LAN-Median** |  |  |  |  |  |  |  |  |  |  |
| Below the median | 15.7 | 16/10 | 1.00 | - |  | 15.4 | 11/11 | 1.00 | - |  |
| Above or equal the median | 32.4 | 18/9 | 1.56 | (0.75-3.26) | 0.237 | 35.1 | 15/8 | 1.70 | (0.76-3.81) | 0.200 |
| **LAN-Tertiles** |  |  |  |  |  |  |  |  |  |  |
| 1^st^ tertile | 10.5 | 8/9 | 1.00 | - |  | 10.5 | 6/9 | 1.00 | - |  |
| 2^nd^ tertile | 26.5 | 14/4 | 3.07 | (1.20-7.86) | 0.019 | 28.0 | 10/5 | 2.65 | (0.87-8.06) | 0.085 |
| 3^rd^ tertile | 36.2 | 12/6 | 2.72 | (1.01-7.28) | 0.047 | 37.0 | 10/5 | 2.82 | (0.95-8.41) | 0.061 |
| **LAN-Fixed cut-offs** |  |  |  |  |  |  |  |  |  |  |
| <15 | 7.8 | 5/6 | 1.00 | - |  | 6.2 | 5/6 | 1.00 | - |  |
| ≥15; < 30 | 22.2 | 14/7 | 2.37 | (0.77-7.28) | 0.130 | 22.2 | 10/7 | 1.82 | (0.56-5.91) | 0.318 |
| ≥ 30 | 35.3 | 15/6 | 4.01 | (1.25-12.84) | 0.020 | 35.4 | 12/6 | 3.22 | (0.97-10.72) | 0.056 |

**Supplementary Table S12.** Risk of dementia according to increasing exposure to outdoor artificial light at night (LAN) using 2014 Visible Infrared Imaging Radiometer Suite (VIIRS) data, using both linear increase and categories based on median (50th), tertiles, and fixed cutoffs of nighttime luminance exposure (LAN) in nW/cm^2^/sr, in a multivariable analysis stratified by sex and adjusted by age, and educational attainment, further adjusted by particulate matter concentrations. Analysis considering as outcome any dementia, and Alzheimer’s dementia only (with exclusion of other dementia cases ab initio). HR: hazard ratio, CI: confidence interval.

| **2014** |  |  |  | **Any dementia** |  |  |  |  | **AD** |  |
| --- | --- | --- | --- | --- | --- | --- | --- | --- | --- | --- |
| **LAN (nW/cm^2^/sr)** | **50^th^** | **N**  **C+/C-** | **OR** | **(95% CI)** | **P value** | **50^th^** | **N**  **C+/C-** | **OR** | **(95% CI)** | **P value** |
| Linear trend (1-unit increase) |  |  | 1.04 | (1.00-1.08) | 0.037 | - |  | 1.04 | (1.00-1.08) | 0.074 |
| Linear trend (10-unit increase) |  |  | 1.48 | (1.02-2.13) | 0.037 | - |  | 1.46 | (0.96-2.20) | 0.074 |
| **LAN-Median** |  |  |  |  |  |  |  |  |  |  |
| Below the median | 15.7 | 16/10 | 1.00 | - |  | 15.4 | 11/11 | 1.00 | - |  |
| Above or equal the median | 32.4 | 18/9 | 1.52 | (0.67-3.42) | 0.313 | 35.1 | 15/8 | 1.70 | (0.69-4.17) | 0.245 |
| **LAN-Tertiles** |  |  |  |  |  |  |  |  |  |  |
| 1^st^ tertile | 10.5 | 8/9 | 1.00 | - |  | 1.05 | 6/9 | 1.00 | - |  |
| 2^nd^ tertile | 26.5 | 14/4 | 3.57 | (1.28-9.96) | 0.015 | 28.0 | 10/5 | 3.13 | (0.93-10.51) | 0.065 |
| 3^rd^ tertile | 36.2 | 12/6 | 3.08 | (1.08-8.78) | 0.036 | 37.0 | 10/5 | 3.29 | (1.01-10.75) | 0.049 |
| **LAN-Fixed cut-offs** |  |  |  |  |  |  |  |  |  |  |
| <15 | 7.8 | 5/6 | 1.00 | - |  | 6.2 | 4/6 | 1.00 | - |  |
| ≥15; < 30 | 22.2 | 14/7 | 2.82 | (0.82-9.65) | 0.099 | 22.5 | 10/7 | 2.13 | (0.57-7.91) | 0.261 |
| ≥ 30 | 35.3 | 15/6 | 4.80 | (1.32-17.41) | 0.017 | 35.4 | 12/6 | 3.80 | (0.98-14.83) | 0.054 |

**Supplementary Table S13.** Risk of dementia according to increasing exposure to outdoor artificial light at night (LAN) using 2014 Visible Infrared Imaging Radiometer Suite (VIIRS) data, using both linear increase and categories based on median (50th), tertiles, and fixed cutoffs of nighttime luminance exposure (LAN) in nW/cm^2^/sr, in a multivariable analysis stratified by sex and adjusted by age, and educational attainment, and further adjusted by smoking status. Analysis considering as outcome any dementia, and Alzheimer’s dementia only (with exclusion of other dementia cases ab initio). HR: hazard ratio, CI: confidence interval.

| **2014** |  |  |  | **Any dementia** |  |  |  |  | **AD** |  |
| --- | --- | --- | --- | --- | --- | --- | --- | --- | --- | --- |
| **LAN (nW/cm^2^/sr)** | **50^th^** | **N**  **C+/C-** | **OR** | **(95% CI)** | **P value** | **50^th^** | **N**  **C+/C-** | **OR** | **(95% CI)** | **P value** |
| Linear trend (1-unit increase) | - |  | 1.03 | (1.00-1.07) | 0.043 | - |  | 1.03 | (0.99-1.07) | 0.102 |
| Linear trend (10-unit increase) | - |  | 1.40 | (1.01-1.94) | 0.043 | - |  | 1.35 | (0.94-1.94) | 0.102 |
| **LAN-Median** |  |  |  |  |  |  |  |  |  |  |
| Below the median | 15.7 | 16/10 | 1.00 | - |  | 15.4 | 11/11 | 1.00 | - |  |
| Above or equal the median | 32.4 | 18/9 | 1.53 | (0.72-3.22) | 0.268 | 35.1 | 15/8 | 1.63 | (0.71-3.76) | 0.252 |
| **LAN-Tertiles** |  |  |  |  |  |  |  |  |  |  |
| 1^st^ tertile | 10.5 | 8/9 | 1.00 | - |  | 10.5 | 6/9 | 1.00 | - |  |
| 2^nd^ tertile | 26.5 | 14/4 | 3.28 | (1.22-8.76) | 0.018 | 28.0 | 10/5 | 2.68 | (0.83-8.63) | 0.099 |
| 3^rd^ tertile | 36.2 | 12/6 | 2.82 | (1.03-7.71) | 0.043 | 37.0 | 10/5 | 2.85 | (0.92-8.82) | 0.069 |
| **LAN-Fixed cut-offs** |  |  |  |  |  |  |  |  |  |  |
| <15 | 7.8 | 5/6 | 1.00 | - |  | 6.2 | 4/6 | 1.00 | - |  |
| ≥15; < 30 | 22.2 | 14/7 | 2.38 | (0.75-7.53) | 0.139 | 22.2 | 10/7 | 1.76 | (0.52-5.97) | 0.366 |
| ≥ 30 | 35.3 | 15/6 | 4.02 | (1.22-13.28) | 0.022 | 35.4 | 12/6 | 3.11 | (0.90-10.77) | 0.074 |

**Supplementary Table S14.** Risk of dementia according to increasing exposure to outdoor artificial light at night (LAN) using 2014 Visible Infrared Imaging Radiometer Suite (VIIRS) data, using both linear increase and categories based on median (50th), tertiles, and fixed cutoffs of nighttime luminance exposure (LAN) in nW/cm^2^/sr, in a multivariable analysis stratified by sex and adjusted by age, and educational attainment, and further adjusted by chronic obstructive pulmonary disease. Analysis considering as outcome any dementia, and Alzheimer’s dementia only (with exclusion of other dementia cases ab initio). HR: hazard ratio, CI: confidence interval.

| **2014** |  |  |  | **Any dementia** |  |  |  |  | **AD** |  |
| --- | --- | --- | --- | --- | --- | --- | --- | --- | --- | --- |
| **LAN (nW/cm^2^/sr)** | **50^th^** | **N**  **C+/C-** | **OR** | **(95% CI)** | **P value** | **50^th^** | **N**  **C+/C-** | **OR** | **(95% CI)** | **P value** |
| Linear trend (1-unit increase) | - |  | 1.04 | (1.00-1.07) | 0.032 | - |  | 1.03 | (0.99-1.07) | 0.094 |
| Linear trend (10-unit increase) | - |  | 1.43 | (1.03-1.97) | 0.032 | - |  | 1.35 | (0.95-1.92) | 0.094 |
| **LAN-Median** |  |  |  |  |  |  |  |  |  |  |
| Below the median | 15.7 | 16/10 | 1.00 | - |  | 15.4 | 11/11 | 1.00 | - |  |
| Above or equal the median | 32.4 | 18/9 | 1.72 | (0.80-3.69) | 0.161 | 35.1 | 15/8 | 1.57 | (0.69-3.56) | 0.283 |
| **LAN-Tertiles** |  |  |  |  |  |  |  |  |  |  |
| 1^st^ tertile | 10.5 | 8/9 | 1.00 | - |  | 10.5 | 6/9 | 1.00 | - |  |
| 2^nd^ tertile | 26.5 | 14/4 | 2.81 | (1.07-7.38) | 0.036 | 28.0 | 10/5 | 2.33 | (0.75-7.22) | 0.143 |
| 3^rd^ tertile | 36.2 | 12/6 | 2.77 | (1.03-7.43) | 0.043 | 37.0 | 10/5 | 2.80 | (0.94-8.38) | 0.065 |
| **LAN-Fixed cut-offs** |  |  |  |  |  |  |  |  |  |  |
| <15 | 7.8 | 5/6 | 1.00 | - |  | 6.2 | 4/6 | 1.00 | - |  |
| ≥15; < 30 | 22.2 | 14/7 | 2.15 | (0.69-6.73) | 0.187 | 22.2 | 10/7 | 1.62 | (0.49-5.34) | 0.432 |
| ≥ 30 | 35.3 | 15/6 | 4.20 | (1.30-13.54) | 0.016 | 35.4 | 12/6 | 3.25 | (0.97-10.90) | 0.056 |

**Supplementary Table S15.** Risk of dementia according to increasing exposure to outdoor artificial light at night (LAN) using 2014 Visible Infrared Imaging Radiometer Suite (VIIRS) data, using both linear increase and categories based on median (50th), tertiles, and fixed cutoffs of nighttime luminance exposure (LAN) in nW/cm^2^/sr, in a multivariable analysis stratified by sex and adjusted by age, and educational attainment, and further adjusted by diabetes. Analysis considering as outcome any dementia, and Alzheimer’s dementia only (with exclusion of other dementia cases ab initio). HR: hazard ratio, CI: confidence interval.

| **2014** |  |  |  | **Any dementia** |  |  |  |  | **AD** |  |
| --- | --- | --- | --- | --- | --- | --- | --- | --- | --- | --- |
| **LAN (nW/cm^2^/sr)** | **50^th^** | **N**  **C+/C-** | **OR** | **(95% CI)** | **P value** | **50^th^** | **N**  **C+/C-** | **OR** | **(95% CI)** | **P value** |
| Linear trend (1-unit increase) | - |  | 1.03 | (1.00-1.07) | 0.038 | - |  | 1.03 | (1.00-1.07) | 0.079 |
| Linear trend (10-unit increase) | - |  | 1.40 | (1.02-1.91) | 0.038 | - |  | 1.36 | (0.96-1.92) | 0.079 |
| **LAN-Median** |  |  |  |  |  |  |  |  |  |  |
| Below the median | 15.7 | 16/10 | 1.00 | - |  | 15.4 | 11/11 | 1.00 | - |  |
| Above or equal the median | 32.4 | 18/9 | 1.58 | (0.75-3.30) | 0.227 | 35.1 | 15/8 | 1.70 | (0.76-3.83) | 0.196 |
| **LAN-Tertiles** |  |  |  |  |  |  |  |  |  |  |
| 1^st^ tertile | 10.5 | 8/9 | 1.00 | - |  | 10.5 | 6/9 | 1.00 | - |  |
| 2^nd^ tertile | 26.5 | 14/4 | 3.05 | (1.19-7.81) | 0.020 | 28.0 | 10/5 | 2.64 | (0.87-8.03) | 0.087 |
| 3^rd^ tertile | 36.2 | 12/6 | 2.75 | (1.02-7.37) | 0.045 | 37.0 | 10/5 | 2.86 | (0.96-8.50) | 0.059 |
| **LAN-Fixed cut-offs** |  |  |  |  |  |  |  |  |  |  |
| <15 | 7.8 | 5/6 | 1.00 | - |  | 6.2 | 4/6 | 1.00 | - |  |
| ≥15; < 30 | 22.2 | 14/7 | 2.32 | (0.75-7.17) | 0.142 | 22.2 | 10/7 | 1.80 | (0.55-5.89) | 0.330 |
| ≥ 30 | 35.3 | 15/6 | 3.99 | (1.25-12.78) | 0.020 | 35.4 | 12/6 | 3.22 | (0.97-10.80) | 0.056 |

**Supplementary Table S16.** Risk of dementia according to increasing exposure to outdoor artificial light at night (LAN) using 2014 Visible Infrared Imaging Radiometer Suite (VIIRS) data, using both linear increase and categories based on median (50th), tertiles, and fixed cutoffs of nighttime luminance exposure (LAN) in nW/cm^2^/sr, in a multivariable analysis stratified by sex and adjusted by age, and educational attainment, and further adjusted by apolipoprotein E ε4 genotype status. Analysis considering as outcome any dementia, and Alzheimer’s dementia only (with exclusion of other dementia cases ab initio). HR: hazard ratio, CI: confidence interval.

| **2014** |  |  |  | **Any dementia** |  |  |  |  | **AD** |  |
| --- | --- | --- | --- | --- | --- | --- | --- | --- | --- | --- |
| **LAN (nW/cm^2^/sr)** | **50^th^** | **N**  **C+/C-** | **OR** | **(95% CI)** | **P value** | **50^th^** | **N**  **C+/C-** | **OR** | **(95% CI)** | **P value** |
| Linear trend (1-unit increase) | - |  | 1.02 | (0.99-1.06) | 0.240 | - |  | 1.01 | (0.97-1.06) | 0.528 |
| Linear trend (10-unit increase) | - |  | 1.25 | (0.86-1.80) | 0.240 | - |  | 1.14 | (0.76-1.13) | 0.528 |
| **LAN-Median** |  |  |  |  |  |  |  |  |  |  |
| Below the median | 14.5 | 11/7 | 1.00 | - |  | 13.2 | 7/8 | 1.00 | - |  |
| Above or equal the median | 32.0 | 14/6 | 1.64 | (0.68-3.96) | 0.273 | 33.5 | 11/5 | 1.69 | (0.61-4.67) | 0.313 |
| **LAN-Tertiles** |  |  |  |  |  |  |  |  |  |  |
| 1^st^ tertile | 11.3 | 7/7 | 1.00 | - |  | 11.3 | 5/7 | 1.00 | - |  |
| 2^nd^ tertile | 28.2 | 10/2 | 2.94 | (0.98-8.77) | 0.053 | 28.6 | 7/3 | 2.12 | (0.59-7.65) | 0.251 |
| 3^rd^ tertile | 37.2 | 8/4 | 2.06 | (0.66-6.41) | 0.212 | 37.3 | 6/3 | 1.77 | (0.48-6.53) | 0.392 |
| **LAN-Fixed cut-offs** |  |  |  |  |  |  |  |  |  |  |
| <15 | 8.3 | 5/4 | 1.00 | - |  | 8.4 | 4/4 | 1.00 | - |  |
| ≥15; < 30 | 22.1 | 9/5 | 1.46 | (0.53-4.92) | 0.540 | 24.0 | 6/5 | 0.79 | (0.19-3.24) | 0.741 |
| ≥ 30 | 35.3 | 11/4 | 2.66 | (0.78-9.04) | 0.118 | 36.2 | 8/4 | 1.69 | (0.44-6.50) | 0.449 |

**Supplementary Table S17.** Risk of dementia according to increasing exposure to outdoor artificial light at night (LAN) using annual 2014 Visible Infrared Imaging Radiometer Suite (VIIRS) data, using both linear increase and categories based on median (50th), tertiles, and fixed cutoffs of nighttime luminance exposure (LAN) in nW/cm^2^/sr, in a multivariable analysis stratified by sex and adjusted by age, and educational attainment, and restricted to non-smokers. Analysis considering as outcome any dementia, and Alzheimer’s dementia only (with exclusion of other dementia cases ab initio). HR: hazard ratio, CI: confidence interval.

| **2014** |  |  |  | **Any dementia** |  |  |  |  | **AD** |  |
| --- | --- | --- | --- | --- | --- | --- | --- | --- | --- | --- |
| **LAN (nW/cm^2^/sr)** | **50^th^** | **N**  **C+/C-** | **OR** | **(95% CI)** | **P value** | **50^th^** | **N**  **C+/C-** | **OR** | **(95% CI)** | **P value** |
| Linear trend (1-unit increase) | - |  | 1.04 | (1.00-1.08) | 0.030 | - |  | 1.04 | (1.00-1.08) | 0.073 |
| Linear trend (10-unit increase) | - |  | 1.48 | (1.04-3.10) | 0.030 | - |  | 1.42 | (0.97-2.10) | 0.073 |
| **LAN-Median** |  |  |  |  |  |  |  |  |  |  |
| Below the median | 15.0 | 13/10 | 1.00 | - |  | 13.2 | 9/10 | 1.00 | - |  |
| Above or equal the median | 32.4 | 15/8 | 1.84 | (0.81-4.18) | 0.148 | 33.8 | 12/8 | 1.81 | (0.72-4.50) | 0.205 |
| **LAN-Tertiles** |  |  |  |  |  |  |  |  |  |  |
| 1^st^ tertile | 8.4 | 8/7 | 1.00 | - |  | 8.4 | 6/7 | 1.00 | - |  |
| 2^nd^ tertile | 26.5 | 9/7 | 2.03 | (0.74-5.55) | 0.168 | 27.9 | 6/7 | 1.53 | (0.46-5.08) | 0.485 |
| 3^rd^ tertile | 35.4 | 11/4 | 2.81 | (0.96-8.18) | 0.058 | 35.4 | 9/4 | 2.58 | (0.80-8.26) | 0.111 |
| **LAN-Fixed cut-offs** |  |  |  |  |  |  |  |  |  |  |
| <15 | 7.8 | 5/6 | 1.00 | - |  | 6.2 | 4/6 | 1.00 | - |  |
| ≥15; < 30 | 21.2 | 9/7 | 1.96 | (0.60-6.46) | 0.268 | 21.7 | 6/7 | 1.36 | (0.37-4.97) | 0.640 |
| ≥ 30 | 35.3 | 14/5 | 4.70 | (1.33-16.55) | 0.016 | 35.3 | 11/5 | 3.54 | (0.97-12.90) | 0.055 |

**Supplementary Table S18.** Risk of dementia according to increasing exposure to outdoor artificial light at night (LAN) using annual 2014 Visible Infrared Imaging Radiometer Suite (VIIRS) data, using both linear increase and categories based on median (50th), tertiles, and fixed cutoffs of nighttime luminance exposure (LAN) in nW/cm^2^/sr, in a multivariable analysis stratified by sex and adjusted by age, and educational attainment, Analysis excluding subjects with diagnosis within 12 months. Analysis considering as outcome any dementia, and Alzheimer’s dementia only (with exclusion of other dementia cases ab initio). HR: hazard ratio, CI: confidence interval.

| **2014** |  |  |  | **Any dementia** |  |  |  |  | **AD** |  |
| --- | --- | --- | --- | --- | --- | --- | --- | --- | --- | --- |
| **LAN (nW/cm^2^/sr)** | **50^th^** | **N**  **C+/C-** | **OR** | **(95% CI)** | **P value** | **50^th^** | **N**  **C+/C-** | **OR** | **(95% CI)** | **P value** |
| Linear trend (1-unit increase) | - |  | 1.03 | (1.00-1.07) | 0.045 | - |  | 1.03 | (0.99-1.07) | 0.094 |
| Linear trend (10-unit increase) | - |  | 1.39 | (1.01-1.91) | 0.045 | - |  | 1.35 | (0.95-1.92) | 0.094 |
| **LAN-Median** |  |  |  |  |  |  |  |  |  |  |
| Below the median | 15.7 | 10/15 | 1.00 | - |  | 15.0 | 11/10 | 1.00 | - |  |
| Above or equal the median | 32.4 | 18/8 | 1.55 | (0.75-3.22) | 0.237 | 33.8 | 14/8 | 1.43 | (0.62-3.29) | 0.406 |
| **LAN-Tertiles** |  |  |  |  |  |  |  |  |  |  |
| 1^st^ tertile | 10.5 | 9/8 | 1.00 | - |  | 9.4 | 6/8 | 1.00 | - |  |
| 2^nd^ tertile | 25.0 | 13/4 | 2.87 | (1.11-7.44) | 0.030 | 27.9 | 9/6 | 2.10 | (0.71-6.24) | 0.182 |
| 3^rd^ tertile | 37.0 | 12/5 | 2.71 | (1.01-7.29) | 0.048 | 37.2 | 10/4 | 2.69 | (0.90-8.03) | 0.075 |
| **LAN-Fixed cut-offs** |  |  |  |  |  |  |  |  |  |  |
| <15 | 7.8 | 5/6 | 1.00 | - |  | 6.2 | 6/4 | 1.00 | - |  |
| ≥15; < 30 | 22.0 | 13/7 | 2.20 | (0.71-6.80) | 0.172 | 22.0 | 9/7 | 1.62 | (0.49-5.34) | 0.432 |
| ≥ 30 | 35.3 | 15/5 | 4.03 | (1.25-12.99) | 0.019 | 35.3 | 12/5 | 3.25 | (0.97-10.90) | 0.056 |

**Supplementary Figure S1.** Flowchart of the study population. AD, Alzheimer’s dementia; CSF, cerebrospinal fluid; FTD, frontotemporal dementia; LBD, Lewy body dementia; MCI, mild cognitive impairment; VaD, Vascular dementia.

**
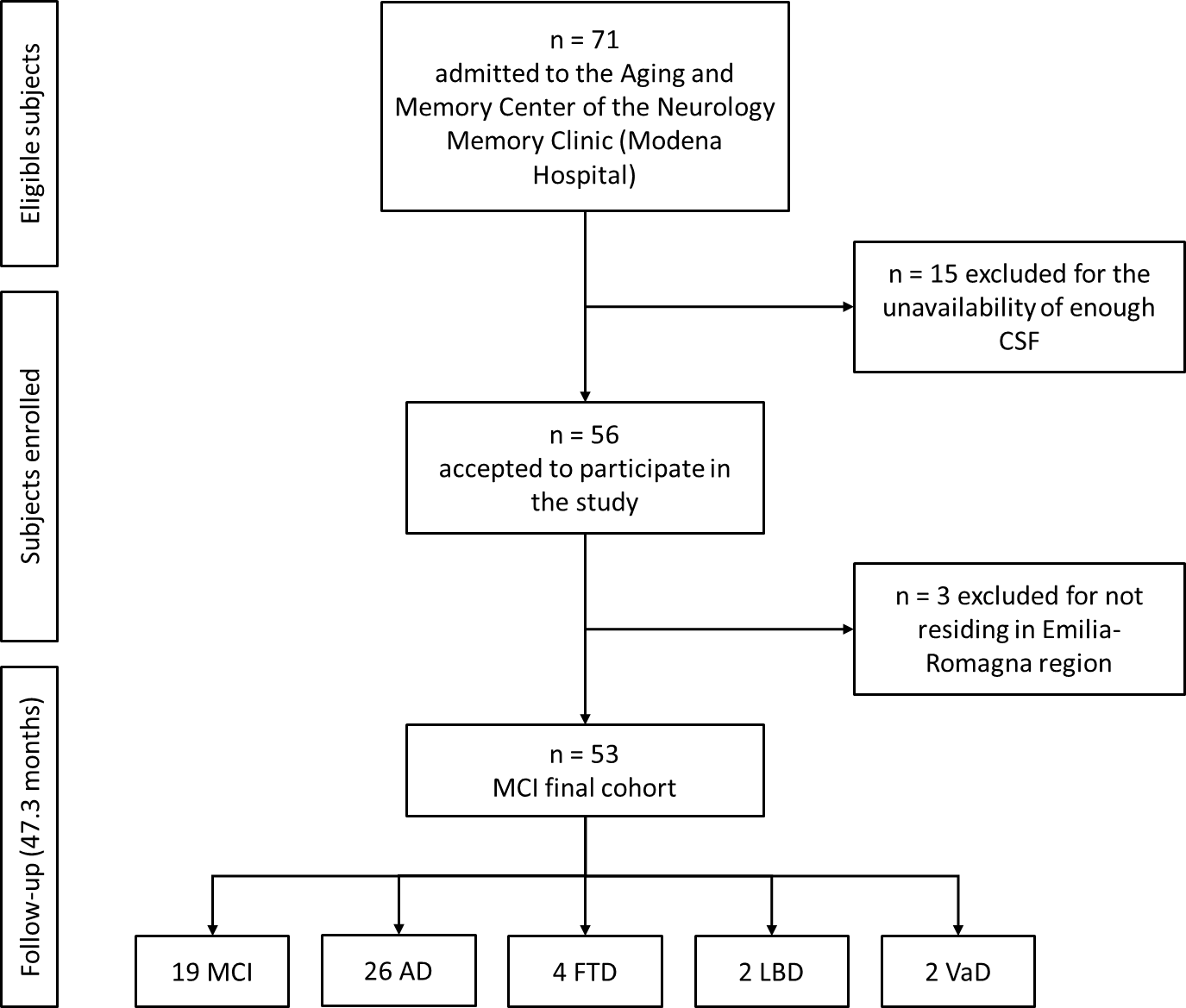
**

**Supplementary Figure S2.** Map of light at night in the Emilia-Romagna region from 2014 to 2022 annual Visible Infrared Imaging Radiometer Suite (VIIRS) data.

**
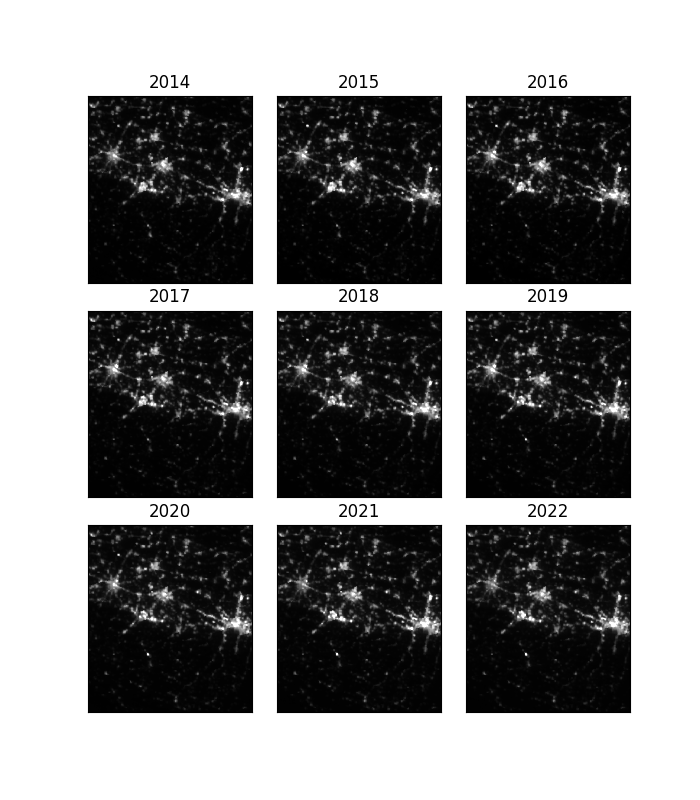
**

**Supplementary Figure S3.** Scatter plot matrix showing correlation of annual average Visible Infrared Imaging Radiometer Suite (VIIRS) data across study years from 2014 to 2022 and average for the entire period 2014-2022.

**Supplementary Figure S4.** Spline correlation analysis between outdoor artificial light at night (LAN) exposure and levels of cerebrospinal biomarkers, i.e. beta-amyloid, total tau protein (t-tau), phosphorylated tau-protein (p-tau), adjusted by traffic-related particulate matter concentrations. Light and dark red dots indicate subjects who remained MCI and converted to dementia, respectively. The blue line represents spline regression analysis with 95% confidence interval (light blue area). The red continuous line represents the biomarker cut-off values used at the Modena Neuroimmunology laboratory (amyloid Aβ_1-42_: 557 pg/mL; t-tau: 350 pg/mL; p-tau: 62 pg/mL).

**
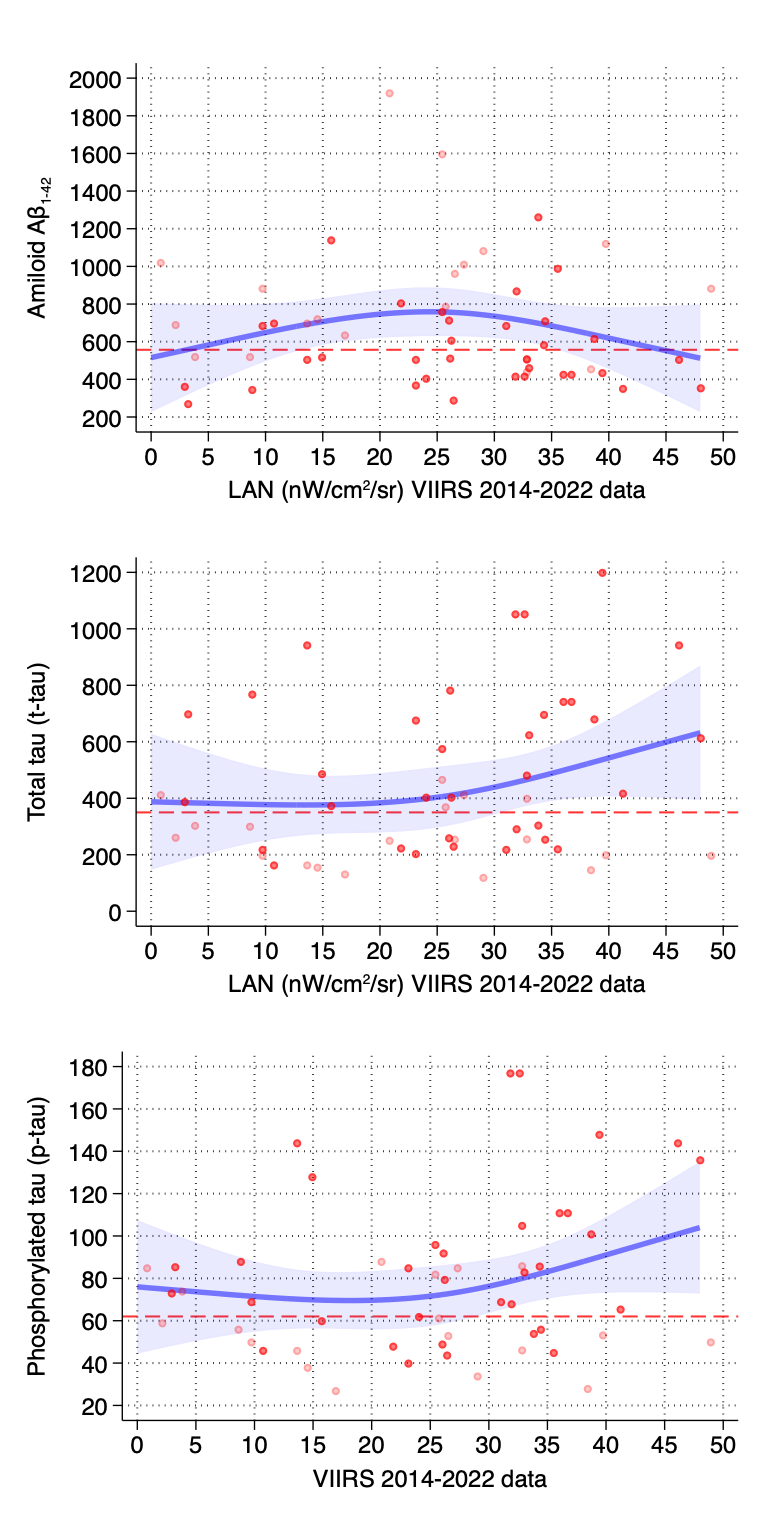
**

**Supplementary Figure S5.** Spline regression analysis for the association between outdoor articificial light at night (LAN) using 9-year average 2014-2022 Visible Infrared Imaging Radiometer Suite (VIIRS) data and risk of developing any type of dementia. The solid line indicates hazard ratio (HR) and the shaded areas the 95% confidence intervals. Analysis considering as outcome any dementia (blue), and Alzheimer’s dementia only (red-with exclusion of other dementia cases ab initio). Analysis stratified by sex and adjusted by age at entry, and education, and further by chronic obstructive pulmonary disease (COPD), diabetes, or APOE4, apolipoprotein E ε4 genotype status (APOE4).


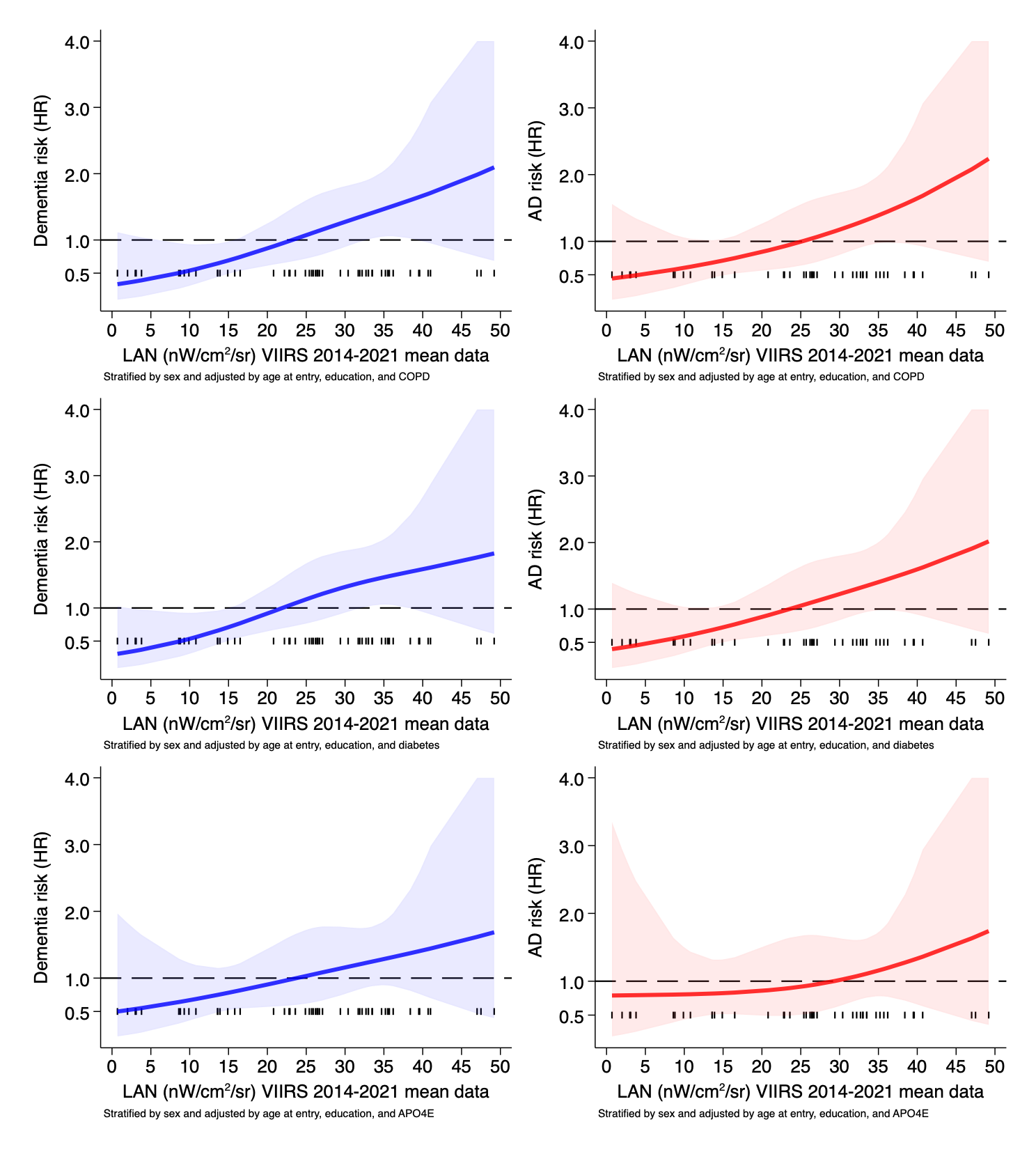


**Supplementary Figure S6.** Spline regression analysis for the association between outdoor articificial light at night (LAN) using 9-year average 2014-2022 Visible Infrared Imaging Radiometer Suite (VIIRS) data and risk of developing any type of dementia. The solid line indicates hazard ratio (HR) and the shaded areas the 95% confidence intervals. Analysis considering as outcome any dementia (blue), and Alzheimer’s dementia only (red-with exclusion of other dementia cases ab initio). Analysis restricted to non-smokers, stratified by sex and adjusted by age at entry, and education, and further by particulate matter, chronic obstructive pulmonary disease (COPD), diabetes, or apolipoprotein E ε4 genotype status (APOE4).


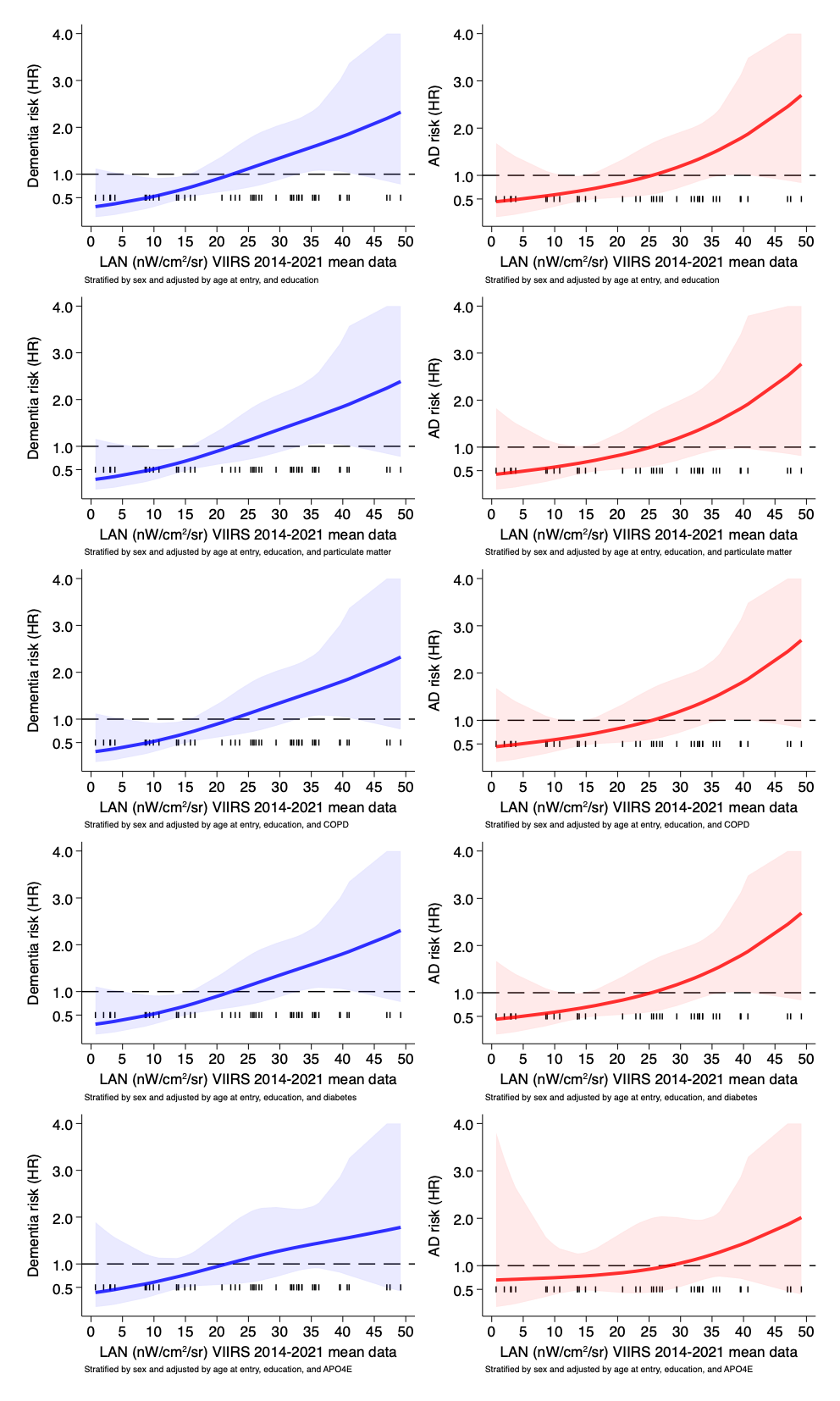


**Supplementary Figure S7.** Spline regression analysis for the association between outdoor articificial light at night (LAN) using 9-year average 2014-2022 Visible Infrared Imaging Radiometer Suite (VIIRS) data and risk of developing any type of dementia. The solid line indicates hazard ratio (HR) and the shaded areas the 95% confidence intervals. Analysis considering as outcome any dementia (blue), and Alzheimer’s dementia only (red-with exclusion of other dementia cases ab initio). Analysis excluding subjects with diagnosis within 12 months. Analysis stratified by sex and adjusted by age at entry, and education, and further by particulate matter, or smoking status.


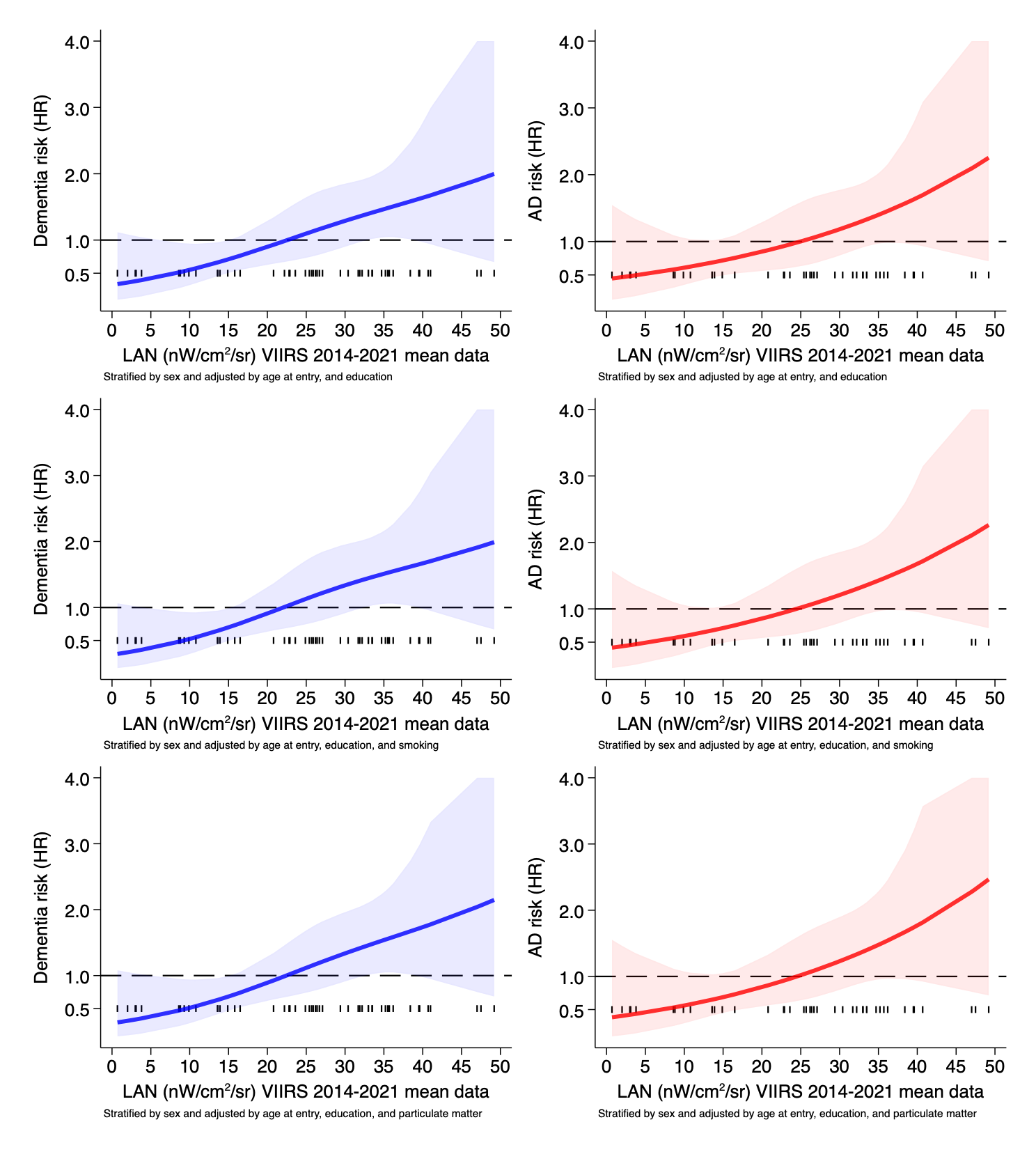


**Supplementary Figure S8.** Spline regression analysis for the association between outdoor articificial light at night (LAN) using 9-year average 2014-2022 Visible Infrared Imaging Radiometer Suite (VIIRS) data and risk of developing any type of dementia. The solid line indicates hazard ratio (HR) and the shaded areas the 95% confidence intervals. Analysis considering as outcome any dementia (blue), and Alzheimer’s dementia only (red-with exclusion of other dementia cases ab initio). Analysis excluding subjects with diagnosis within 12 months. Analysis stratified by sex and adjusted by age at entry, and education, and further by chronic obstructive pulmondary disease (COPD), diabetes or APOE4, apolipoprotein E ε4 genotype status (APOE4).

**
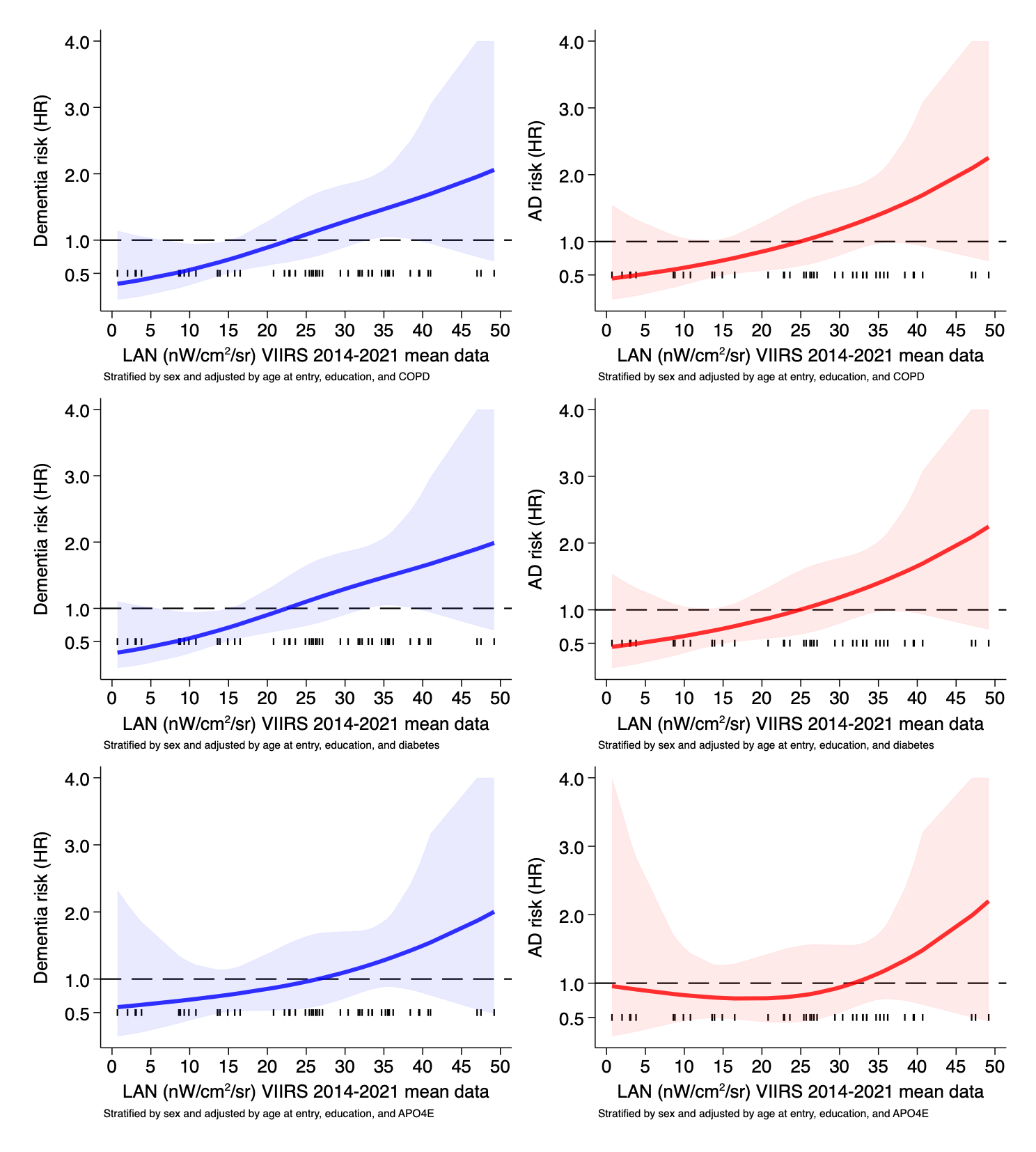
**

**Supplementary Figure S9.** Spline regression analysis for the association between outdoor articificial light at night (LAN) using annual 2014 Visible Infrared Imaging Radiometer Suite (VIIRS) data and risk of developing any type of dementia. The solid line indicates hazard ratio (HR) and the shaded areas the 95% confidence intervals. Analysis considering as outcome any dementia (blue), and Alzheimer’s dementia only (red-with exclusion of other dementia cases ab initio). Analysis stratified by sex and adjusted by age at entry, and education, and further by particulate matter, or smoking status.


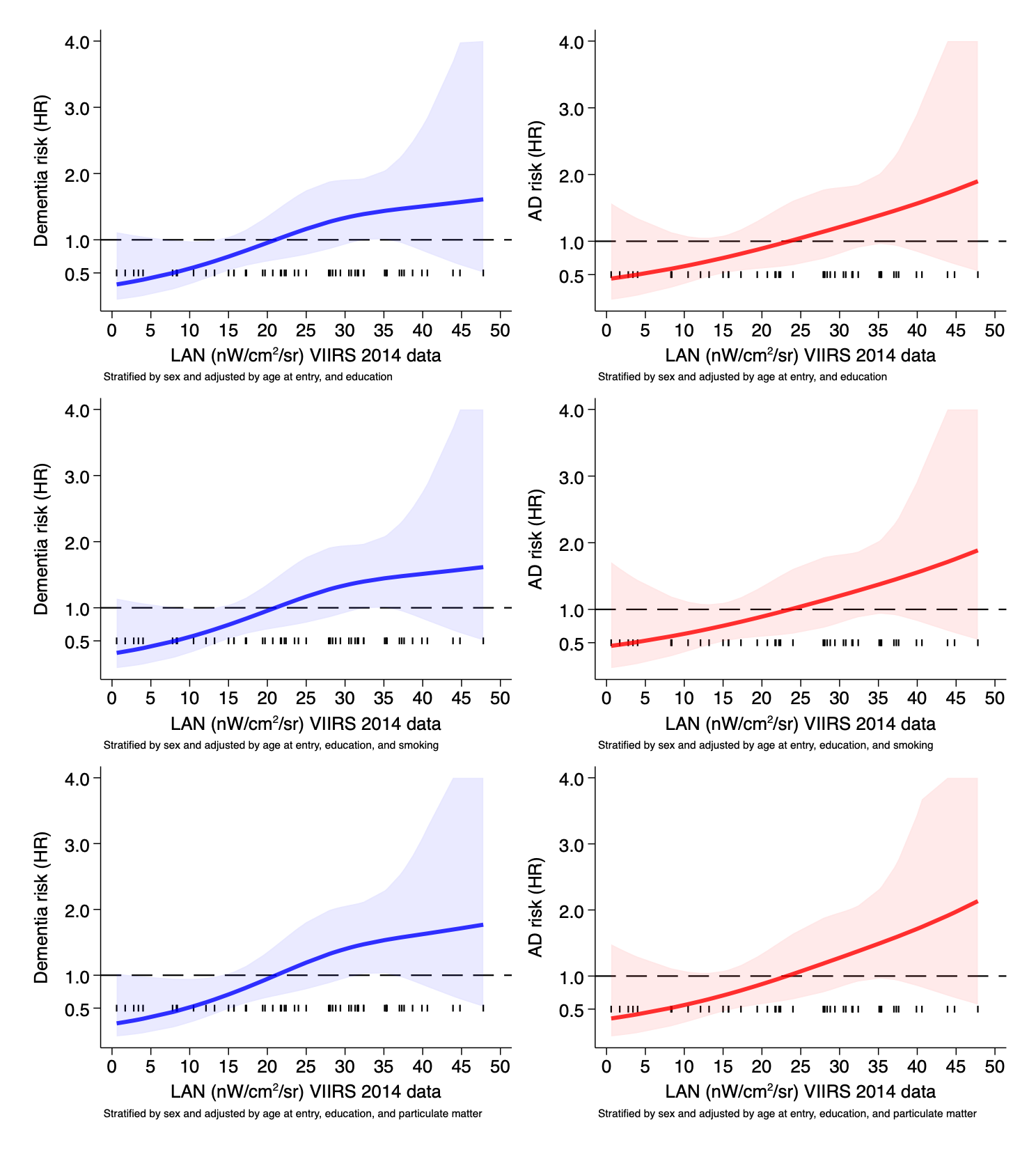


**Supplementary Figure S10.** Spline regression analysis for the association between outdoor articificial light at night (LAN) using annual 2014 Visible Infrared Imaging Radiometer Suite (VIIRS) data and risk of developing any type of dementia. The solid line indicates hazard ratio (HR) and the shaded areas the 95% confidence intervals. Analysis considering as outcome any dementia (blue), and Alzheimer’s dementia only (red-with exclusion of other dementia cases ab initio). Analysis stratified by sex and adjusted by age at entry, and education, and further by chronic obstructive pulmonary disease (COPD), diabetes, or APOE4, apolipoprotein E ε4 genotype status (APOE4).

**
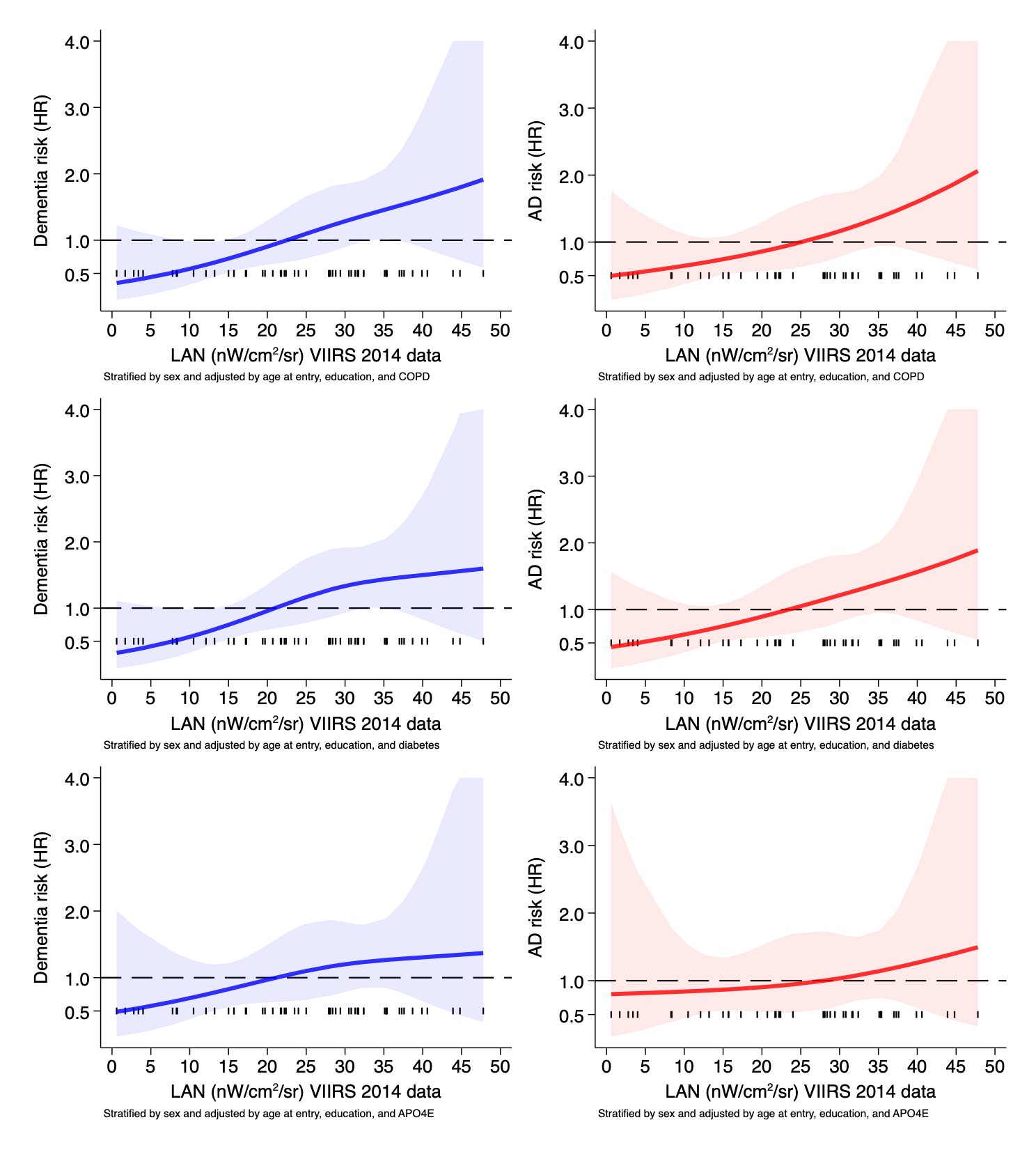
**

**Supplementary Figure S11.** Spline regression analysis for the association between outdoor articificial light at night (LAN) using 2014 Visible Infrared Imaging Radiometer Suite (VIIRS) data and risk of developing any type of dementia. The solid line indicates hazard ratio (HR) and the shaded areas the 95% confidence intervals. Analysis considering as outcome any dementia (blue), and Alzheimer’s dementia only (red-with exclusion of other dementia cases ab initio). Analysis restricted to non-smokers and stratified by sex and adjusted by age at entry, and education, and further by particulate matter, chronic obstructive pulmonary disease (COPD), diabetes, or apolipoprotein E ε4 genotype status (APOE4).


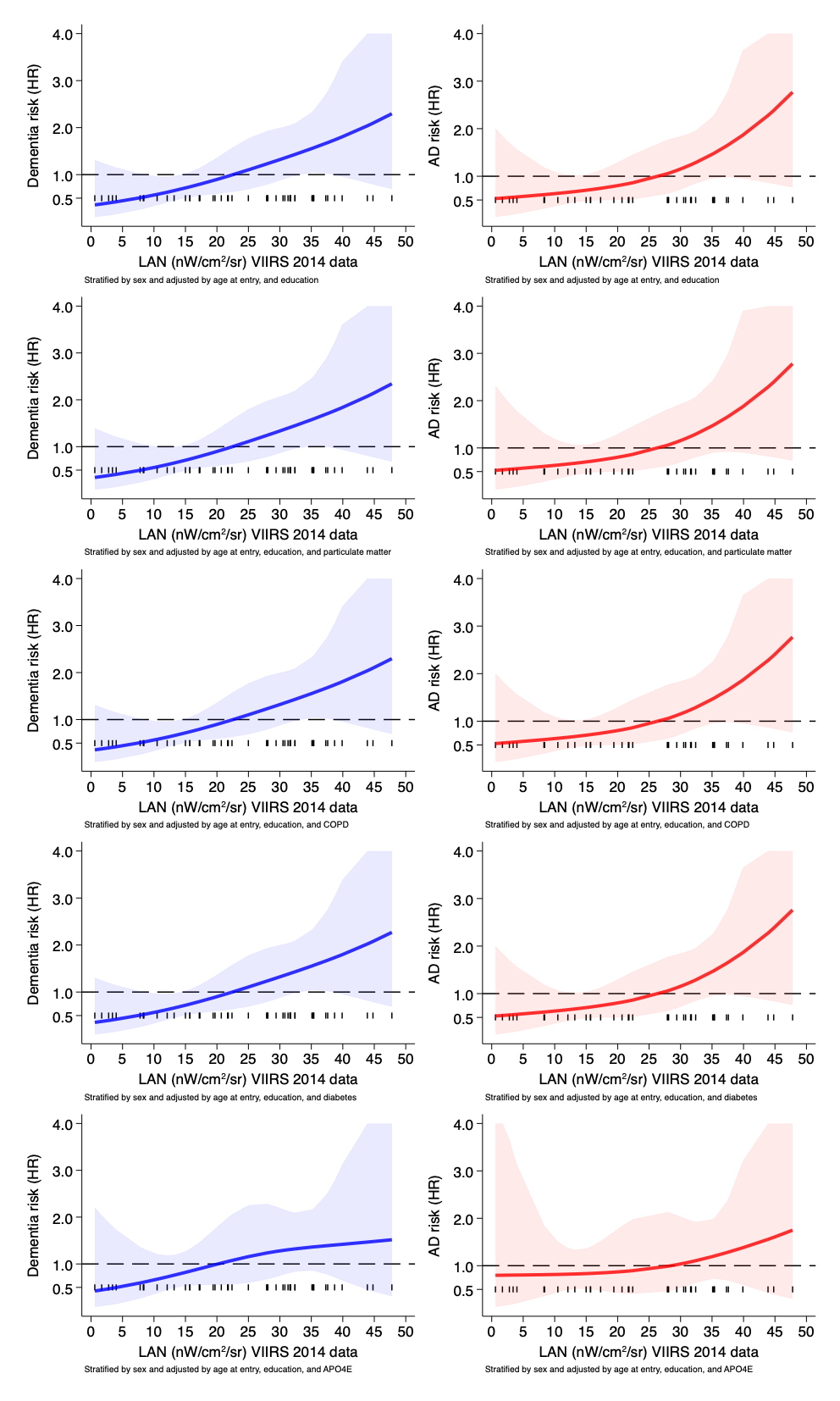


**Supplementary Figure S12.** Spline regression analysis for the association between outdoor articificial light at night (LAN) using 2014 Visible Infrared Imaging Radiometer Suite (VIIRS) data and risk of developing any type of dementia. The solid line indicates hazard ratio (HR) and the shaded areas the 95% confidence intervals. Analysis considering as outcome any dementia (blue), and Alzheimer’s dementia only (red-with exclusion of other dementia cases ab initio). Analysis excluding subjects with diagnosis within 12 months. Analysis stratified by sex and adjusted by age at entry, and education, and further by particulate matter, or smoking status.


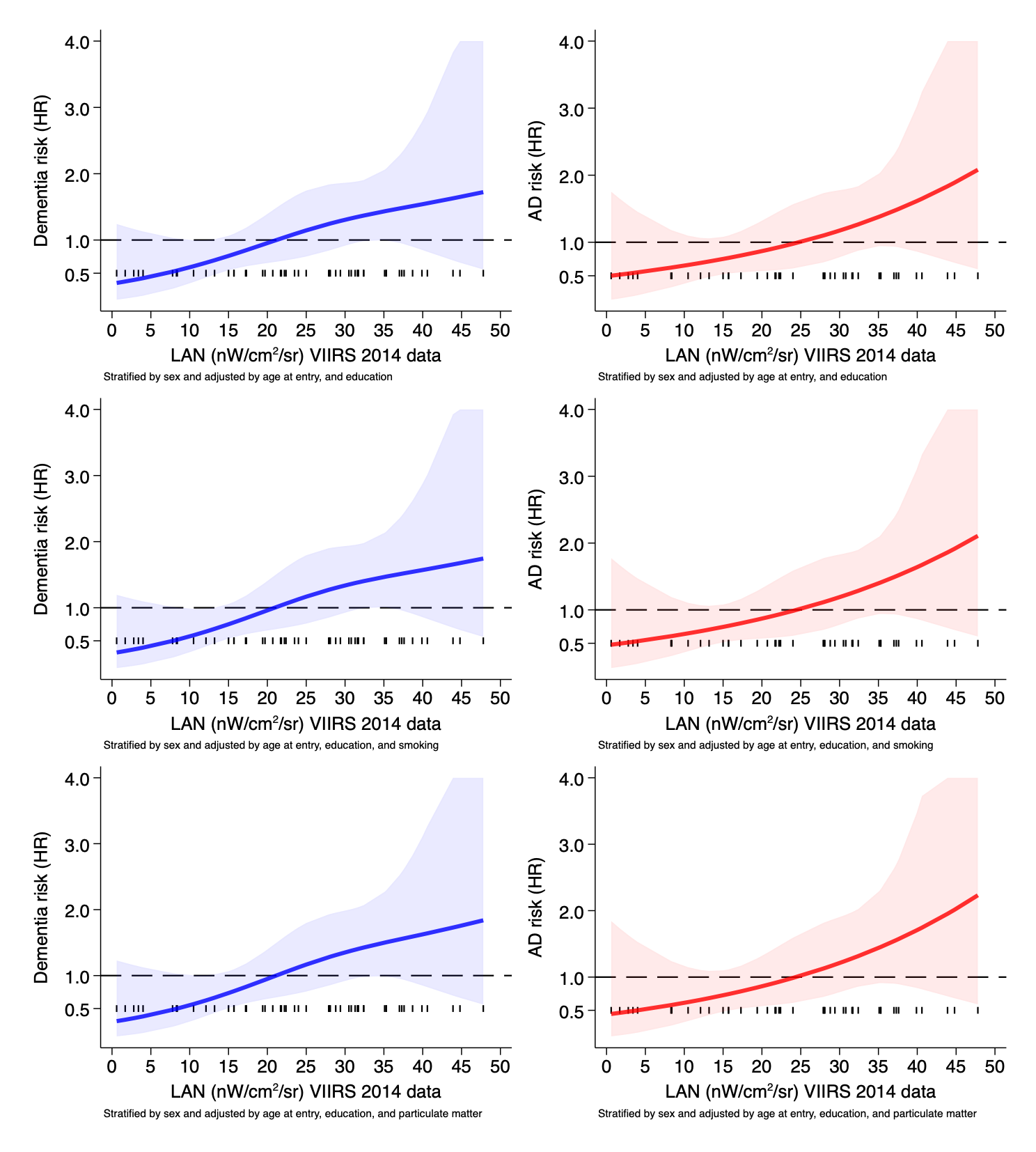


**Supplementary Figure S13.** Spline regression analysis for the association between outdoor articificial light at night (LAN) using 2014 Visible Infrared Imaging Radiometer Suite (VIIRS) data and risk of developing any type of dementia. The solid line indicates hazard ratio (HR) and the shaded areas the 95% confidence intervals. Analysis considering as outcome any dementia (blue), and Alzheimer’s dementia only (red-with exclusion of other dementia cases ab initio). Analysis excluding subjects with diagnosis within 12 months. Analysis stratified by sex and adjusted by age at entry, and education, and further by chronic obstructive pulmonary disease (COPD), diabetes, or apolipoprotein E ε4 genotype status (APOE4).

**
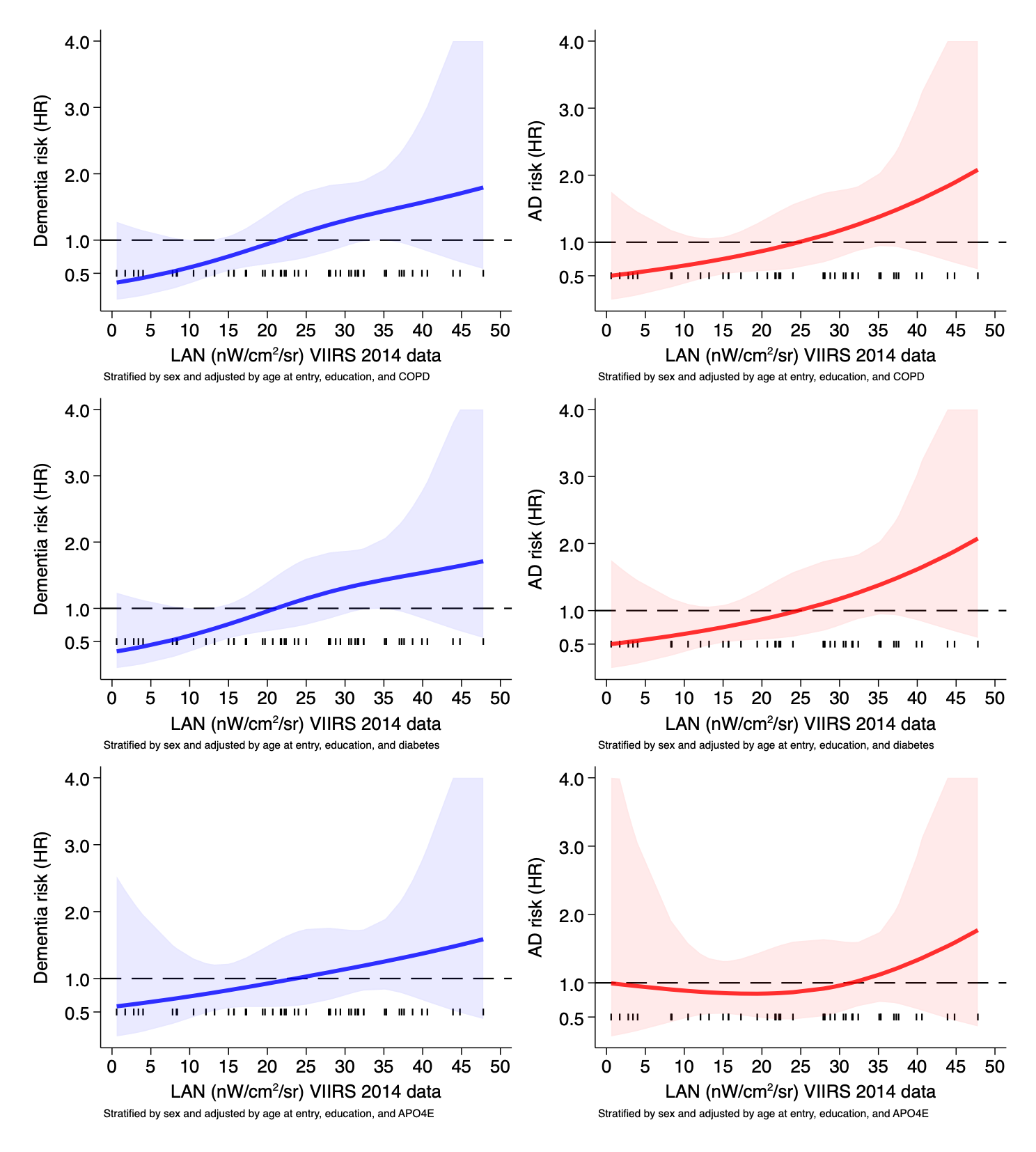
**
